# Advances in Additive Manufacturing Electrochemistry

**DOI:** 10.1021/acs.chemrev.5c00984

**Published:** 2026-06-05

**Authors:** Robert D. Crapnell, Elena Bernalte, Craig E. Banks

**Affiliations:** Faculty of Science and Engineering, 5289 Manchester Metropolitan University, Dalton Building, Chester Street, Manchester M1 5GD, Great Britain

## Abstract

Fused filament fabrication (FFF) has rapidly evolved from a prototyping tool into a powerful platform for electrochemical device innovation. Its accessibility, design freedom, and compatibility with diverse polymers have enabled breakthroughs in sensor architectures, energy storage components, and wearable systems. However, works using currently available commercial filament lack the required conductivity or functionality to create impactful devices. Recent advances in conductive composites, surface functionalization strategies, and multimaterial printing have transformed FFF from a low-resolution technique into a versatile manufacturing approach capable of integrating complex geometries with tailored electrochemical properties with the potential to of achieve real impact. While challenges remain in realizing microscale resolution and consistentency in conductivity between filament batches and in printing across different devices, emerging solutions are pushing the boundaries of what is possible. This review highlights the progress that has positioned FFF at the forefront of additive manufacturing for electrochemistry and outlines the innovations that will drive the next generation of scalable, high-performance devices.

## Graphical abstract

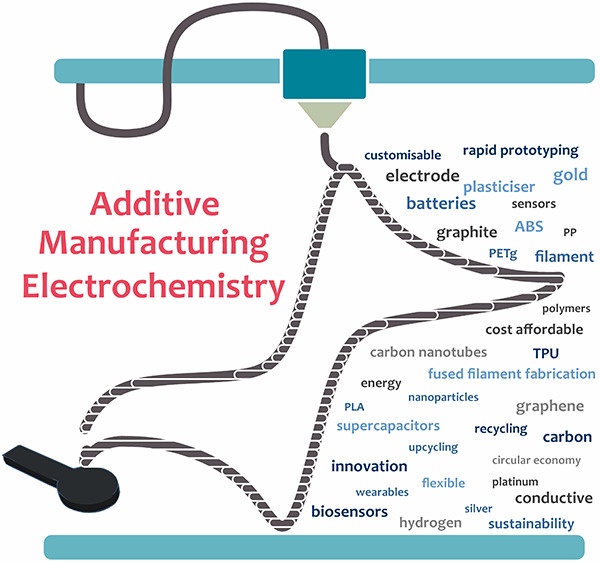

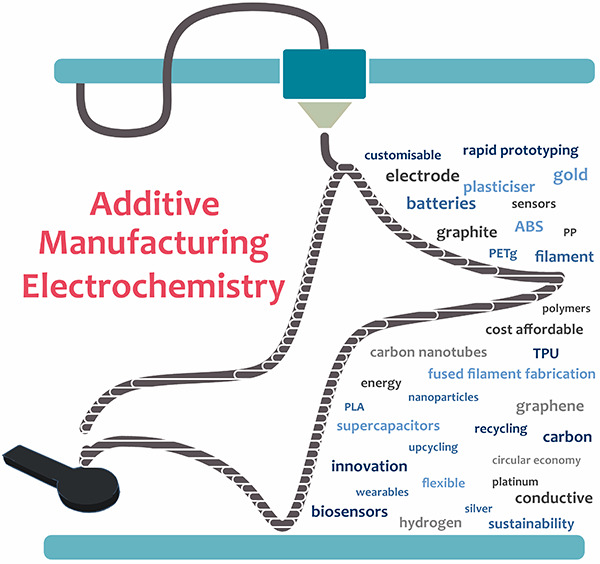

## 1 Introduction

The convergence of additive manufacturing and electrochemistry represents a transformative shift in the way electrochemical devices can be potentially designed, fabricated, and deployed.^1^ Traditionally, electrochemical systems (ranging from sensors to energy storage devices) have relied on rigid, planar and often labor-intensive fabrication methods. Although effective, these approaches have imposed significant limitations on design flexibility, customization and scalability, which particularly hinders the transition from laboratory prototypes into real-world impacting applications. Compared to traditional methods, such as subtractive or formative, additive manufacturing, also colloquially referred to as 3D-printing, offers compelling solutions to the challenges hindering impact. Additive manufacturing is a broad term encompassing many manufacturing methods that allow for the construction of 3-dimensional objects from digital design files in a layer-by-layer fashion. In such a way, additive manufacturing techniques introduce a new paradigm of on-demand, waste-minimized, and highly customizable complex geometries.^2,3^ The global additive manufacturing market has been valued at $44.62 billion in 2024, and is projected to reach $290.9 billion by 2033, with a compound annual growth rate (CAGR) of 23.16%.^4^ This growth is driven by the adoption of additive manufacturing from many industries, including aerospace, automotive, and medical sectors, with a key reason being the increased adoption of advanced materials.^5^ Among the various additive manufactured techniques, Fused Filament Fabrication (FFF), also commonly referred to as Fused Deposition Modeling (FDM), has emerged as a particularly attractive method due to its affordability, accessibility, and compatibility with a wide range of polymeric and composite materials.

The fusion of additive manufacturing and electrochemistry stems from several synergetic advantages, including: (1) Unprecedented design freedom, allowing for the creation of intricate electrode architectures, including nested, porous, overhanging, and multimaterial structures that are otherwise impossible with traditional methods; (2) Rapid prototyping and iteration, which allows researchers to quickly fabricate and test new designs in house, accelerating innovation cycles and project development; (3) Customization and personalization, allowing researchers to tailor their devices to specific applications, environments, or user needs; (4) Decentralized manufacturing, whereby digital files can be shared globally, enabling local production of devices with no requirement for costly centralized infrastructure; (5) Sustainability through the inherent ultralow waste generation of additive manufacturing and its suitability for incorporation into potential circular economies.

Despite the potential of additive manufacturing electrochemistry, there are significant bottlenecks that still need to be overcome before widespread adoption and high-performance implementation can be achieved. Within FFF for example, one of the most accessible and widely used additive manufacturing techniques, the electrochemical performance of commercially available conductive filaments remains a major limitation.^6^ These filaments often suffer from poor electrical conductivity, low electrochemical activity, and inconsistent reproducibility, which restricts their utility in sensitive or high-performance applications.^7^ Moreover, the limited availability and range of conductive filaments on the market narrows the scope of materials researchers can work with, often forcing compromises in mechanical integrity, printability, or chemical compatibility.^8^ Even when conductive filaments are available, their composition is proprietary, making it difficult to tailor or optimize them for specific electrochemical functions. The layer-by-layer nature of FFF also introduces anisotropy in conductivity and mechanical properties, which can affect device performance and reliability.^9^ These challenges have prompted a wave of innovation focused on bespoke filament development, where researchers engineer their own materials, often incorporating high loadings of conductive fillers, to achieve superior performance.^10^ However, this approach introduces its own complexities, including dispersion challenges, printability trade-offs, and scaling difficulties. Additionally, postprocessing techniques such as electrochemical activation, thermal annealing, or chemical treatments are often required to enhance surface properties and improve electrochemical behavior, adding further steps to the fabrication workflow.^11,12^

Despite these bottlenecks, the positives of additive manufacturing electrochemistry are compelling. The ability to fabricate customized, complex geometries with minimal waste and rapid turnaround times opens new avenues for device design and prototyping. Additive manufacturing enables decentralized manufacturing, allowing researchers and practitioners to produce devices locally from digital files, which is particularly valuable in resource-limited or remote settings. The integration of recycled materials and sustainable design principles also aligns additive manufacturing electrochemistry with broader goals in green chemistry and circular economy. Furthermore, the interdisciplinary nature of this field, bridging materials science, engineering, chemistry, and sustainability, makes it a fertile ground for innovation. As bespoke materials improve and printing technologies evolve, additive manufacturing electrochemistry is poised to deliver next-generation electrochemical devices that are precise, portable, scalable, and environmentally responsible.

This review aims to provide a comprehensive and critical overview of the rapidly expanding field of additive manufacturing electrochemistry. As research in this area continues to grow rapidly in scale and diversity, a consolidated analysis is necessary to bring clarity to the materials, methods, applications, and emerging trends that shape the discipline. Our objective is to collate the current state of knowledge, identify technological and methodological gaps, and outline the opportunities that will define the next stages of development in this field. To achieve this, we undertook a systematic and exhaustive survey of the literature, using targeted keyword searches across major academic databases to capture all relevant contributions spanning fundamental studies, fabrication strategies, material innovations, and device-level applications. Through this structured approach, we aim to equip researchers across disciplines with a coherent framework and critical insights needed to harness the full potential of additive manufacturing in electrochemistry.

## 2 Overview of Additive Manufacturing Technologies

Additive manufacturing encompasses a diverse set of fabrication technologies that build objects layer-by-layer from digital models. The ASTM F42 committee on additive manufacturing technologies has formally classified additive manufacturing into seven distinct process categories, summarized in Figure 1, each defined by the method of material deposition, bonding, and energy input.^13,14^ Each offers unique advantages and limitations in terms of resolution, material compatibility, conductivity, and scalability.

**Figure 1:**
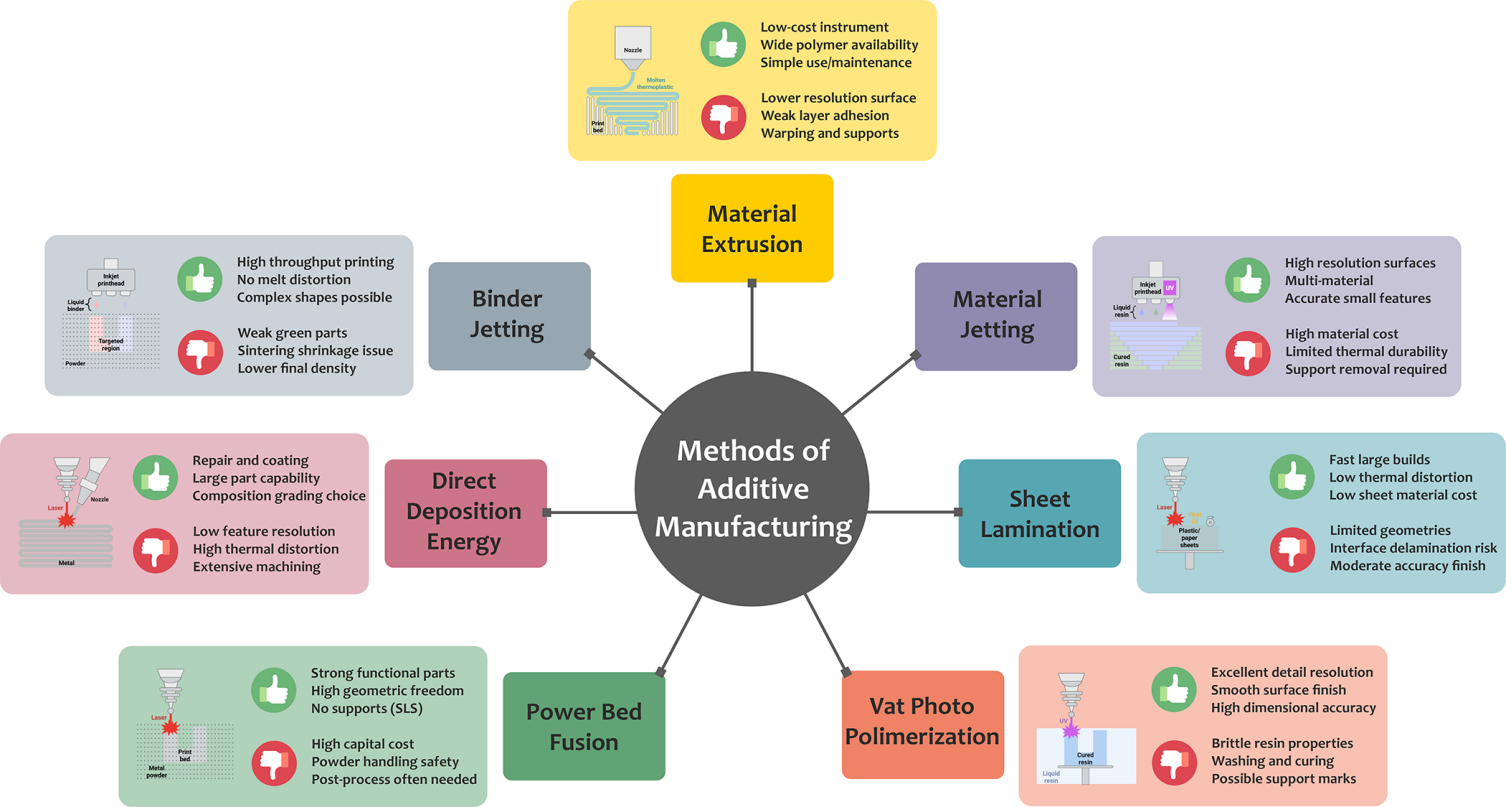
An overview of the seven distinct additive manufacturing process categories outlined by the ASTM F42 committee.

Material extrusion is the most widely adopted additive manufacturing category,^15^ particularly in the form of FFF.^16,17^ This technique involves the extrusion of thermoplastic-based filaments through a heated nozzle, depositing material layer-by-layer to build the final object onto a print bed. Its popularity stems from its affordability, accessibility, and compatibility with a wide range of polymers.^18^ Material extrusion is central to additive manufacturing electrochemistry due to its ease of use and the growing availability of conductive filaments.

Vat photopolymerization encompasses technologies such as stereolithography (SLA) and digital light processing (DLP), which use a light source to selectively cure liquid photopolymer resins. These methods offer high resolution and excellent surface finish, making them attractive for applications requiring fine detail. While traditionally limited to insulating materials, recent advances in conductive resin formulations have opened new possibilities for electrochemical device fabrication using vat photopolymerization.^19,20^ Although possible, the classical route of incorporating carbon-based conductive fillers is severely limited due to the requirements for light facilitated polymerization.^21^ On the other hand, powder bed fusion includes techniques like selective laser sintering (SLS) and electron beam melting (EBM), which use focused energy sources to fuse powder particles into solid structures. These methods are particularly suited for metals and ceramics, offering high mechanical strength and thermal stability.^22,23^ In electrochemistry, powder bed fusion holds promise for fabricating robust, high-performance electrodes and current collectors, although the complexity and cost of these systems can be prohibitive for routine laboratory use. There are some examples of electrochemical studies using powder bed fusion, but they focus on characterizing commercial metal-based powders.^24–26^ SLS is seen as a potential avenue for additive manufacturing electrochemistry due to its synergy with some polymeric materials already reported for FFF, such as poly­(propylene) (PP)^27^ and thermoplastic poly­(urethane) (TPU).^28^

Binder jetting involves the selective deposition of a liquid binder onto a powder bed, followed by postprocessing steps such as sintering or infiltration. This technique enables the fabrication of porous structures and is compatible with a range of materials, including metals and ceramics.^29^ While less common in electrochemical applications, binder jetting offers potential for producing porous electrodes with high surface area and tunable architecture.^30,31^ Alternatively, material jetting operates by depositing droplets of build material in a controlled manner, often followed by curing with UV light. This category offers high resolution and multimaterial printing capabilities, making it suitable for applications requiring precise spatial control of functional materials.^32,33^ However, its use in electrochemistry is currently limited by the availability of suitable conductive inks and the relatively low throughput of the process.

Sheet lamination builds objects by bonding and cutting sheets of material, typically using adhesives or ultrasonic welding.^34,35^ Although rarely used in electrochemical device fabrication, sheet lamination may offer opportunities for integrating conductive layers or creating hybrid structures when combined with other techniques. Directed energy deposition (DED) uses focused thermal energy, such as a laser, electron beam, or plasma arc, to melt and deposit material simultaneously. This technique is primarily used for metals and is well-suited for large-scale or repair applications.^36,37^ In the context of electrochemistry, DED could enable the fabrication of custom current collectors or structural components for energy systems, though its resolution and material constraints limit its use for fine-featured devices.^38,39^

Among the seven additive manufacturing categories, material extrusion, and specifically FFF, has emerged as the most widely adopted technique within the field of additive manufacturing electrochemistry.^40–43^ Its accessibility, affordability, and compatibility with a growing range of functional materials make it particularly attractive for researchers aiming to fabricate custom electrochemical devices. In the following section, we delve deeper into the principles, materials, and electrochemical relevance of FFF, with a particular emphasis on the use of commercially available conductive filaments and the innovations driving bespoke filament development.

### 2.1 Fused Filament Fabrication (FFF)

FFF is a widely adopted additive manufacturing technique that constructs three-dimensional objects through the layer-by-layer deposition of thermoplastic materials. This method has gained significant traction in both industrial, academic and home settings due to its cost-effectiveness, accessibility, and versatility in fabricating complex geometries. Note that FFF and FDM (Fused Deposition Modeling) describe the same additive manufacturing process, but FDM is a trademark, while FFF is the open, generic term and we use FFF throughout.

The FFF process begins with a digital 3D model, typically designed using computer-aided design (CAD) software and exported in formats such as STL or 3MF. This model is then processed using slicing software, which converts the geometry into discrete layers and generates machine-readable G-code instructions. These instructions dictate the movement of the printer’s components and the extrusion path of the material.^44^ At the core of an FFF system is the extruder assembly, which comprises a cold end and a hot end. The cold end is responsible for feeding the thermoplastic filament into the hot end. The hot end contains a heating element and a nozzle, where the filament is melted at temperatures typically ranging from 180 to 260 °C, depending on the material. The molten polymer is then extruded through the nozzle and deposited onto a build platform in a predetermined pattern.^45^ The build platform, or print bed, plays a critical role in ensuring proper adhesion of the initial layers and minimizing warping, particularly for materials prone to thermal contraction.^46–48^ As each layer is deposited, the print head or build platform moves incrementally along the Z-axis to allow the next layer to be printed. The extruded material rapidly cools and solidifies, bonding to the previous layer to form a cohesive structure. Cooling fans are often employed to accelerate solidification and improve dimensional accuracy.^49,50^ Several parameters influence the quality and performance of FFF-printed parts, including layer height, print speed, nozzle temperature, bed temperature, and infill density.^51^ Poor print quality can lead to voids, which has been shown to significantly affect the mechanical strength of parts, a key consideration with many works suffering from poor reproducibility between filament batches and across different printers.^52–54^ Additionally, complex geometries or overhanging features may necessitate the use of support structures, which are removed postprinting. Postprocessing steps such as sanding, annealing, or chemical smoothing may be employed to enhance surface finish or mechanical properties.^55,56^

Recent advancements in FFF technology have introduced multiextruder systems,^57,58^ for example Figure 2, which significantly enhance the versatility and efficiency of the printing process. Initially, multimaterial printing was achieved through manual filament changes or single-nozzle systems like the Prusa Multi Material Unit (MMU), which sequentially fed different filaments into a single hot end. While effective, this approach often required extensive purging between material changes to prevent cross-contamination, leading to substantial material waste and increased print times. To address these limitations, dual-extruder systems such as the RAISE3D E2 have been developed, allowing simultaneous use of two independent print heads. This configuration enables cleaner transitions between materials or colors,^59^ and supports the use of dedicated support materials, such as water-soluble polymers,^60,61^ without the need for excessive purging. Further innovations include tool-changing platforms like the E3D ToolChanger, which accommodates up to four independent tool heads, and the Prusa XL, which supports up to five. These systems physically swap entire extruder assemblies, virtually eliminating the need for purging between material changes and significantly reducing waste. Such capabilities are particularly advantageous in research environments where multimaterial functionality, including conductive/nonconductive interfaces or chemically resistant support structures, is essential for fabricating complex chemical devices or sensor platforms.^62,63^

**Figure 2:**
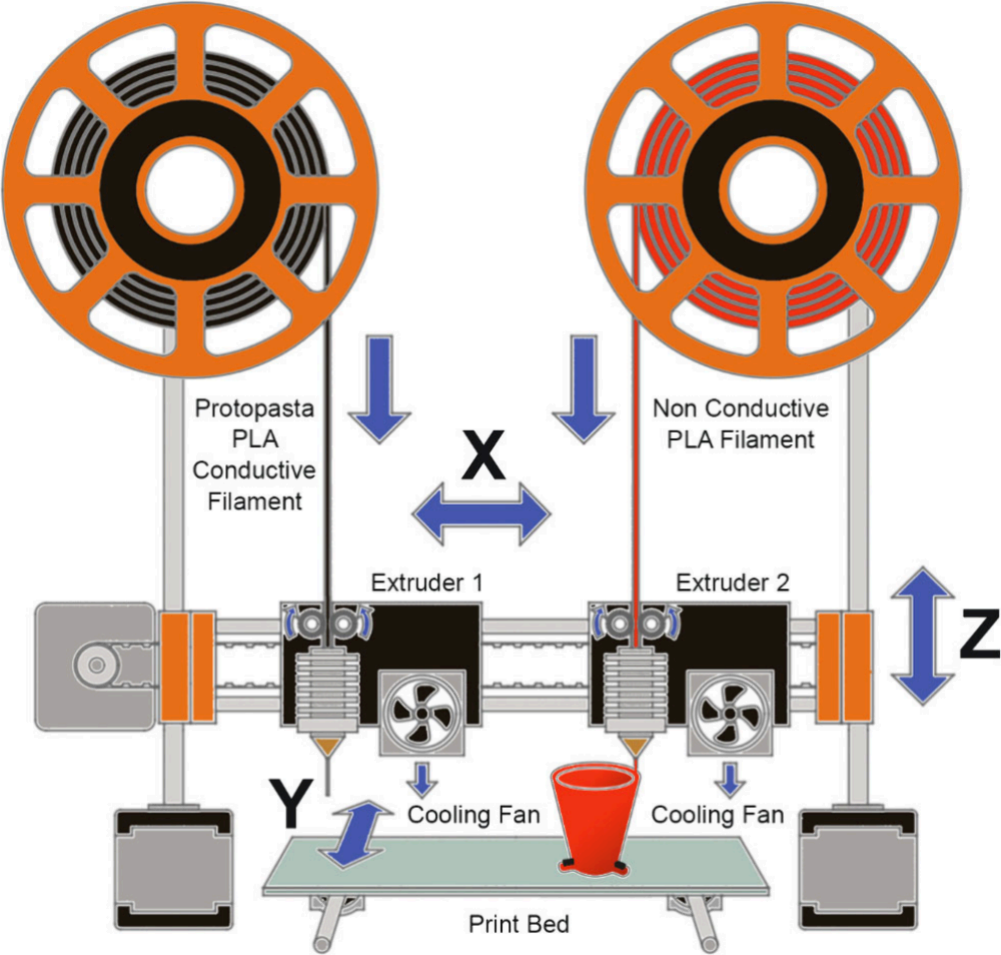
Schematic overview of FFF printing on a dual-extruder system. Reproduced from ref 64. Copyright American Chemical Society 2021, License CC BY-NC-ND 4.0.

Despite its advantages, FFF has limitations. The layer-by-layer nature of the process can result in anisotropic mechanical properties^65–67^ and relatively rough surface finishes compared to other additive manufactured techniques like SLA or selective laser sintering SLS.^68,69^ Furthermore, the mechanical strength and thermal stability of FFF-printed parts are constrained by the properties of the thermoplastic feedstock.^70,71^ Nevertheless, the method remains a cornerstone of rapid prototyping and is increasingly being explored for functional applications in materials science, including the fabrication of electrochemical sensors, microfluidic devices, and customized laboratory equipment. Figure 3 summarizes some key advancements of FFF-based additive manufacturing in the field of electrochemistry, which we will now move to explore, beginning with commercially available filaments.

**Figure 3:**
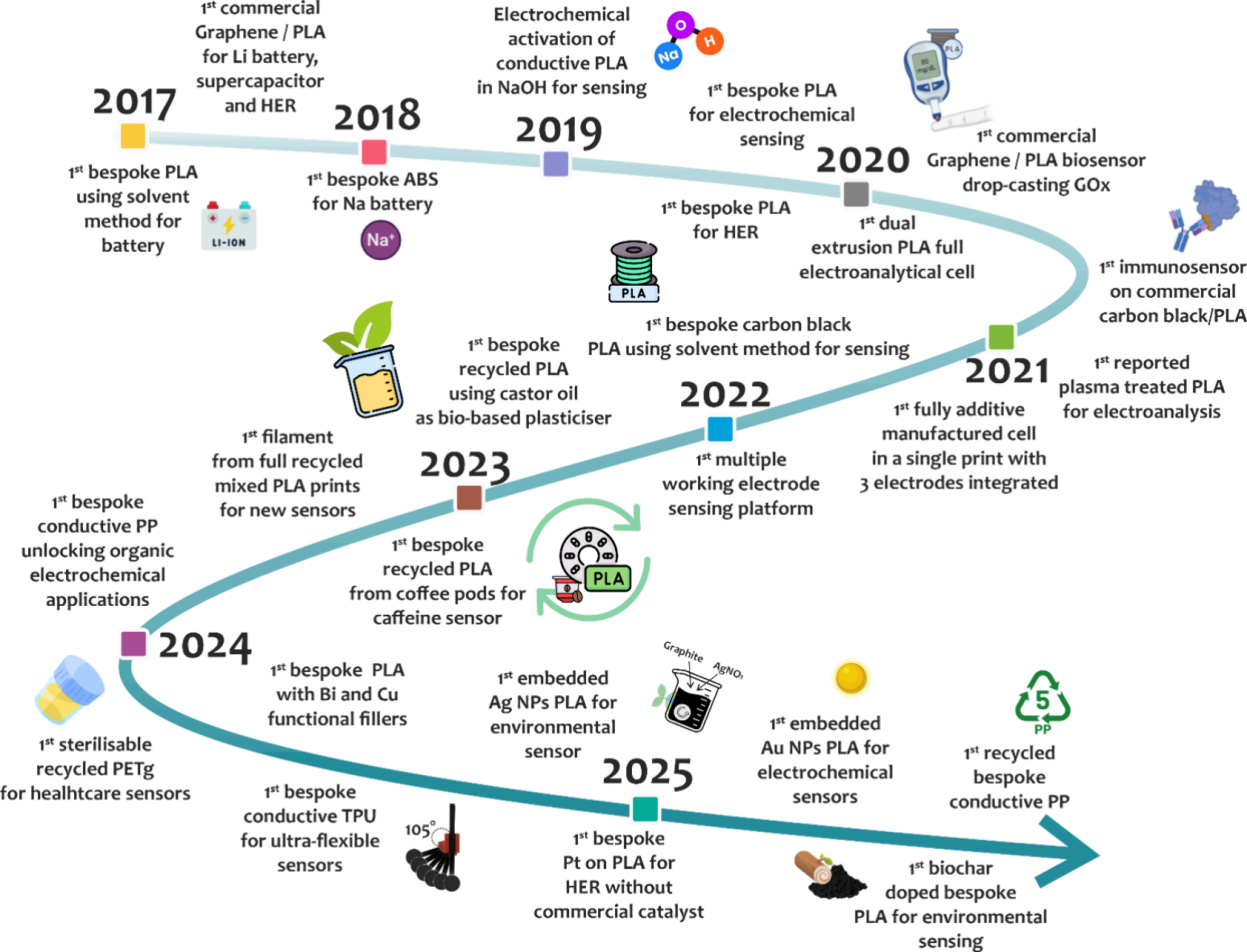
Overview of some key developments within FFF-based additive manufacturing electrochemistry. Information collected from refs 27, 64, and 72–91 .

### 2.2 Commercial Conductive Filaments

The growing popularity of FFF within the electrochemical research community is largely driven by its cost-effectiveness and accessibility. The relatively low capital investment required for desktop FFF printers, combined with the affordability of thermoplastic filaments, has made this technology particularly attractive for research laboratories.^92^ Moreover, the widespread availability of materials, ranging from standard polymers to functional composites, has enabled researchers to rapidly prototype and fabricate custom components without the need for specialized infrastructure.^93^ Among these materials, commercially available conductive filaments have garnered significant attention for their potential in producing electrically functional structures. The majority of commercially available conductive filaments are based on polylactic acid (PLA), a thermoplastic derived from renewable resources such as corn starch or sugar cane. PLA is often marketed as biodegradable; however, this property is only realized under specific industrial composting conditions that involve elevated temperatures, controlled humidity, and microbial activity. In practice, such infrastructure is not widely available, and PLA-based waste typically persists in conventional waste streams. Despite this limitation, PLA remains the dominant base polymer in FFF due to its favorable printing characteristics. It exhibits low warping, good dimensional stability, and a relatively low extrusion temperature (typically 180–220 °C), making it compatible with a wide range of desktop 3D printers.

Exploration into the use of these filaments began with poly­(lactic acid) (PLA) based Black Magic 3D and Protopasta.^94,95^ Both filaments offer ease of printing and good dimensional stability. However, their conductive performance and mechanical characteristics differ due to variations in filler type and loading.^96^ Here it is important to note some key limitations of commercial conductive filaments. First, the proprietary nature of their compositions, which often lack detailed disclosure regarding filler type, concentration, and polymer modifications, as well as potentially containing contaminants.^97^ This opacity can hinder reproducibility and material optimization in research contexts, as well as making true revelations about molecular level interactions and phenomena challenging. Additionally, the availability of these filaments is not guaranteed long-term. For example, Black Magic 3D conductive PLA, one widely used for its graphene-based formulation, has been discontinued, leaving researchers without a direct replacement and highlighting the fragility of relying solely on commercial sources.

As such, Protopasta conductive PLA is by far the most widely used filament. It incorporates carbon black as its conductive filler and reports its composition as PLA resin > 67 wt%, carbon black < 20 wt%, and other polymers < 13 wt%.^94,95^ It has a reported resistance range measured along 10 cm of filament of between 2.0 – 3.5 kΩ, measured using a standard digital multimeter.^95^ It prints reliably at temperatures between 195–225 °C and adheres well to heated beds at 50–60 °C, mechanically it retains the stiffness of PLA, and its surface finish is typically matte and slightly rough due to the particulate nature of the filler. However, its conductivity is limited, making it suitable primarily for low-current applications or capacitive sensing. To overcome the conductive shortcomings of Protopasta, researchers have developed several procedures applied postprint, to improve the performance, typically known as ‘activation’.

### 2.3 Postprint Treatment or “Activation”

Many viable methodologies have been reported in the literature, such as mechanical treatment,^96,98^ heat treatment,^99–101^ chemical treatment,^102–105^ electrochemical treatment,^106–108^ and many more, including combining methods.^11,12,109–112^ The effect of some of the most popular methods can be seen in Figure 4.

**Figure 4:**
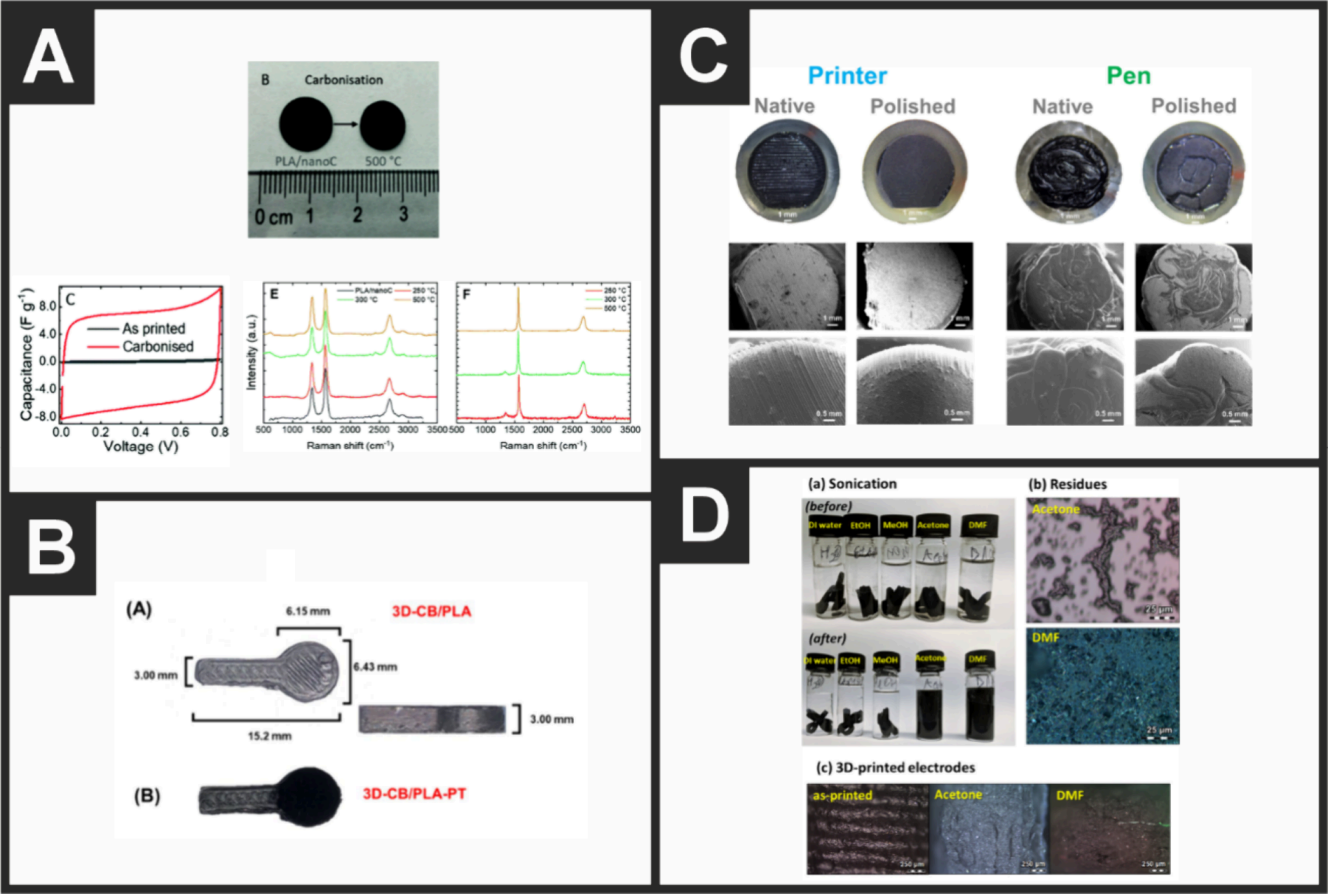
Effects of different activation methodologies on additive manufactured electrodes; (A) carbonisation, reproduced from ref 100, copyright Royal Society of Chemistry 2020; (B) plasma, reproduced from ref 113, available under a CC-BY 4.0 license; (C) mechanical polishing, reproduced from ref 98, copyright Elsevier 2022; (D) solvent activation, reproduced from ref 107, copyright Elsevier 2019.

Mechanical polishing is a straightforward and widely used postprocessing technique. By physically abrading the surface, typically using fine sandpaper, polishing pads, or abrasive slurries, the insulating polymer matrix (e.g., PLA) is selectively removed, exposing the embedded conductive filler. While effective, mechanical polishing requires careful control to avoid damaging the structural integrity of the printed part or introducing surface inconsistencies that may affect reproducibility; although uniquely surface contamination through mechanical action has been used as a sample collection technique.^114^

Heat treatment, particularly thermal annealing or carbonization, subjects printed structures to elevated temperatures, typically in inert atmospheres such as nitrogen or argon, whereby the polymer matrix can be partially or fully decomposed, exposing and restructuring the embedded conductive filler.^100^ This process not only increases the electroactive surface area but can also improve conductivity through graphitization and removal of insulating residues. The carbonization temperature plays a critical role in tuning the final electrode properties, with higher temperatures generally yielding greater capacitance and lower charge transfer resistance. However, excessive thermal treatment may compromise mechanical integrity or dimensional fidelity, necessitating careful optimization based on the intended application.

Chemical activation involves immersing the printed electrodes in reactive solutions. These treatments etch away portions of the PLA matrix, increasing the exposure of conductive fillers. They are relatively simple to implement but require careful control to avoid overetching or damaging the electrode structure. Solutions such as DMF and NaOH are commonly seen within the literature to these ends.

Electrochemical methods typically involve applying a potential in a suitable electrolyte (e.g., phosphate buffer). This process can clean and activate the surface by removing passivating layers and enhancing the electroactive area. It is often used in combination with chemical pretreatment to further improve performance. In fact, the most commonly used activation seen throughout the literature was reported by Richter et al.,^74^ whereby the electrodes are placed within a solution of NaOH (0.5 M) and treated using chronoamperometry through the application of + 1.4 V for 200 s, followed by application of −1.0 V for 200 s. The electrodes are then thoroughly rinsed with deionized water and dried before further use. The effectiveness of this method has been shown for both commercial and bespoke PLA-based filaments, whereby significant amounts of PLA are removed from the electrode surface exposing greater amounts of conductive material. This is exemplified within Figure 5, which shows SEM micrographs of electrode surfaces before and after this treatment for 10 different PLA filament compositions. This accessibility of the conductive carbon is vital, in particular for sensing applications that look to detect molecules that utilize an inner-sphere redox mechanism.^115^

**Figure 5:**
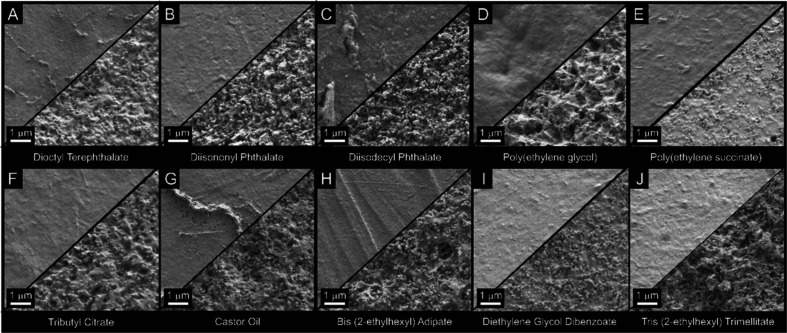
SEM micrographs for electrodes printed from 10 different PLA filament compositions before (top left) and after (bottom right) chemical/electrochemical activation within NaOH (0.5 M) with chronoamperometric application of +1.4 V for 200 s and −1.0 V for 200 s. Reproduced with permission from ref 116. Copyright Royal Society of Chemistry 2025, license CC BY-NC 3.0.

The commercial viability of additive manufactured electrodes is currently significantly hindered due to the requirement of lengthy or complex postprint procedures to obtain functional electrodes. One more recently explored activation method that has the potential to change this is laser^117–119^ and/or plasma treatment.^80,113,120^ Laser ablation and plasma exposure are reagent-free techniques that offer precise and localized activation. These methods remove surface polymer layers and expose conductive pathways without introducing chemical waste. Plasma treatments, in particular, are valued for their reproducibility and environmental friendliness, though they may require specialized equipment. With development of appropriate technologies, it can be envisioned that electrodes could be printed and activated simultaneously with a laser source attached to the print-head or through multi tool systems. This removal of wet chemistry and synergy between two rapid techniques could significantly enhance the viability of additive manufacturing electrochemistry.

From this section it is clear there is a lack of standardized methodologies for postprocessing, activation, and characterization of bespoke filaments. Electrochemical properties are highly sensitive to treatment protocols such as electrochemical activation, solvent exposure, or mechanical polishing, yet these steps are often inconsistently reported. Establishing community-agreed standards for activation procedures, benchmarking electrodes, and reporting conductivity values (such as through four-point probe measurements and full resistivity profiles rather than single-point readings) would greatly enhance cross-study comparability.

### 2.4 Design and Printing Optimization

Almost all of the methods above focus on improving the performance postprint, but work has also been reported on alterations that can be made throughout the printing process to achieve significant improvements in electrochemical performance. If we consider the life cycle of an electrode, it is initially designed on a computer, sliced to create the printable G-code, and then produced on the printer. Due to the rapid prototyping capabilities of additive manufacturing, within the design phase is where significant improvements can be made based on the specific application to be used. It is commonplace within the literature to utilize simple shapes for benchmarking of electrodes, in-particular for bespoke filament, such as ‘lollipops’.^121^ In this design, a disc electrode is printed with a connecting stem, allowing for simple connection to the potentiostat via a crocodile clip. For the applications, however, there is scope to truly exploit the design freedoms of additive manufacturing. For example, through printing different electrode geometries,^78,122–128^ including diverse surface architectures,^129,130^ or the development of full electrochemical cells in a single print.^64,131^ With these designs, it is important to note that conductive filaments are not perfect conductors. Due to the high amount of insulating plastic material, significant resistance is introduced into the systems, which increases significantly depending on the length of the connecting path. It has been shown that through reducing the printed connection length with commercial Protopasta filament from 100 mm to 10 mm, a ∼2.5× improvement in peak-to-peak separation for the common inner-sphere benchmarking probe [Fe­(CN)_6_]^3–^ can be achieved.^132^ It is important to consider that these changes are reflected in the solution resistance (R_s_), measured through electrochemical impedance spectroscopy (EIS), with values of 1.24 ± 0.03 kΩ and 9.07 ± 0.13 kΩ measured for the 10 mm and 100 mm samples, respectively. These issues can potentially be overcome in two ways. First, through the use of near-perfect conductors as connectors, as some have reported,^133^ however, this removes a lot of the design freedoms and innovation that additive manufacturing can provide, as well as introducing additional postprinting steps which hinders commercial viability. Second, through the use of IR compensation, which is commonly used in other areas of electrochemistry,^134–138^ however with no supporting studies currently showing this functioning for additive manufactured electrodes, it remains predominantly unused within the literature.

Once designed, the file is sliced into an appropriate G-code for printing. There are many different slicing software programs available, with many printing companies having their own specific ones alongside their printers. Within this software is where the user can predefine the parameters the printer will follow when producing their electrode. First, we can consider the orientation of the electrode, where works have shown that printing electrodes in a vertical orientation can cause a reduction in the resistivity of the printed parts and lead to an improvement in electrochemical performance for both PLA^139^ and acrylonitrile butadiene styrene (ABS)-based systems,^140^ although it should be noted in these works that the print quality of the final product can vary, especially when printing vertically as some overhanging features are present in certain shapes.

Due to the control afforded by 3D-printers, many other variables are available for tuning.^141^ The layer thickness for example has been tested between 0.1 and 0.4 mm, where it was shown that a lower layer thickness of 0.1 mm produced enhanced electrochemical results.^139^ This was attributed to the more structured conductive pathways formed by the geometrically constrained materials leading to a greater chance of point-to-point conductive contact between layers. The same group progressed to testing the potential impacts of extruder temperature, nozzle diameter and print bed temperature for Protopasta conductive PLA.^142^ Although the nozzle diameter and print bed temperature showed no significant impact on the performance, increasing the extruder temperature from the normal 210–220 °C range for conductive PLA, up to 230–240 °C resulted in improved electrochemical activity.^143^ This was attributed to an increase in surface roughness and reduction in the number of voids seen between each layer. This indicates that it is important to balance the print quality and electrochemical performance, with both being important for a true final product. Additionally, this team has investigated the effect of print speed, comparing electrodes printed between 20 and 100 mm s^–1^, finding that electrodes printed at 60 mm s^–1^ produced the best electrochemical performance in terms of peak current and ΔE_p_.^144^ The majority of this work has been performed on Protopasta conductive PLA, which is the most commonly commercially available conductive filament and is made with only carbon black as a conductive filler. As we will see later, these single filler material filaments are no longer the only ones studied, so work should be done to correlate these findings with filaments utilizing other morphologies.

One other key area that can be manipulated within the slicing software is the infill percentage and the pattern of the infill used in the print. Recent studies have shown that reducing infill below 100% can significantly enhance electrode activity by increasing porosity and improving electrolyte penetration.^145,146^ Lower infill reduces the proportion of insulating polymer within the electrode, thereby exposing a greater fraction of conductive filler and creating more accessible pathways for charge transfer. This structural modification also decreases internal resistance, which is particularly beneficial for applications requiring rapid electron transport and efficient mass transfer. However, the benefits of reduced infill must be balanced against mechanical considerations. Extremely low infill values can compromise structural integrity, leading to fragile electrodes that are unsuitable for practical handling or long-term use and therefore a compromise between enhanced electrochemical performance and sufficient mechanical strength must be found. The way in which a printer produces this infill can vary greatly, as shown in Figure 6.

**Figure 6:**
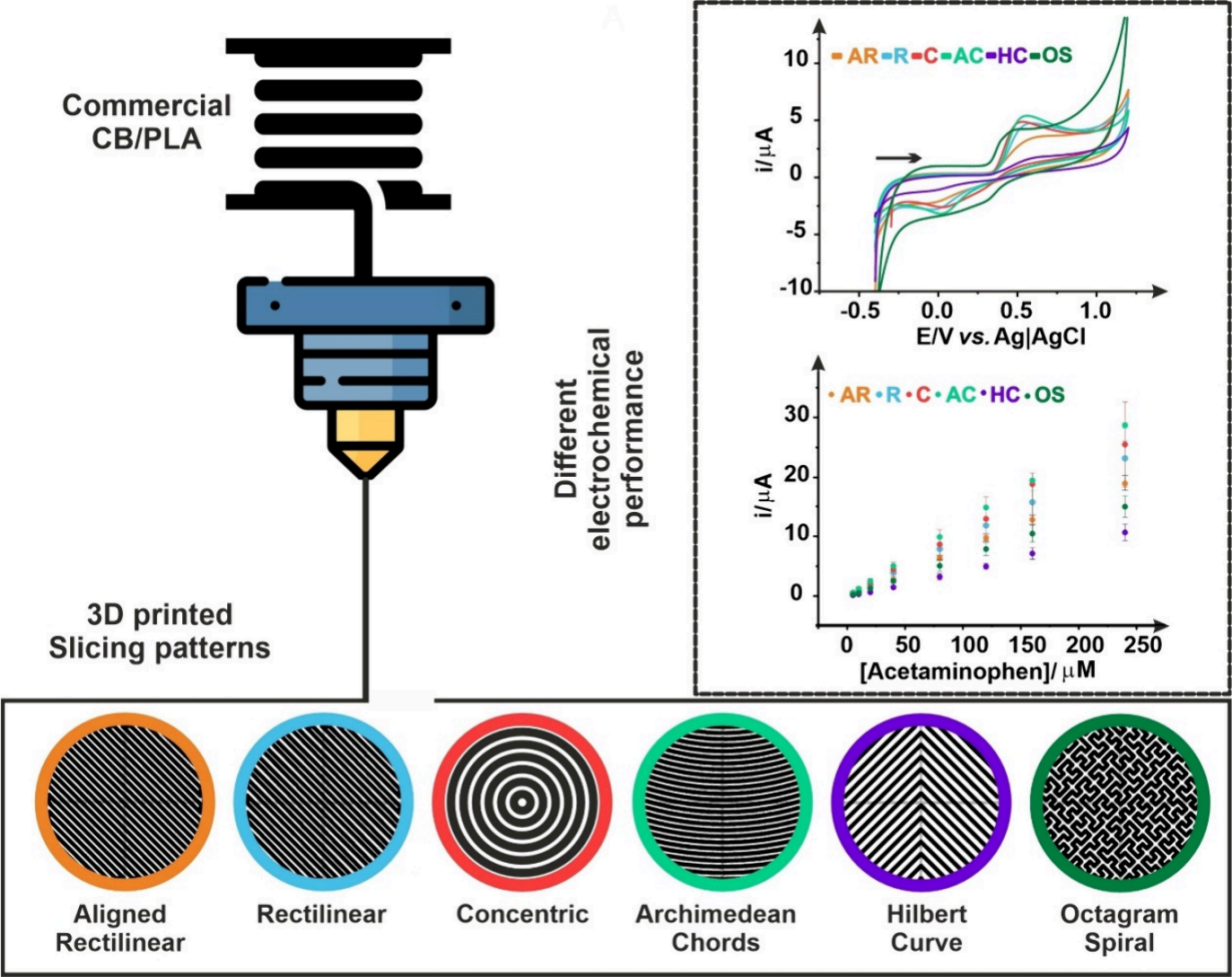
Schematic representation of six different infill patterns and their effect on the peak oxidation current. Reproduced with permission from ref 147. Copyright Wiley 2024, License CC-BY 4.0.

In this work, the authors showed how rectilinear an aligned rectilinear printing patterns produced more consistent electrodes in terms of electrochemical performance, as well as giving excellent print quality. All of these methods, as well as combinations of them have been used to enhance the performance of conductive commercial filaments for electrochemical applications; however, they still fall significantly short of classical electrodes used within electrochemistry such as glassy carbon electrodes (GCE), boron-doped diamond (BDD), and even screen-printed electrodes (SPEs).^148^ Interesting work has recently looked at the utilization of artificial intelligence (AI) for optimizing additive manufacturing conditions for nonconductive work, which could potentially bring enhancements in design precision and efficiency for additive manufacturing electrochemistry in the future.^149^ Fundamentally, this is due to the properties of the filament, which have been designed for hobby printing and the creation of circuits rather than use within electrochemistry. As such, this filament is only appropriate for applications when focusing on other important aspects of additive manufacturing, such as the design freedoms it can offer. To truly embed additively manufactured electrodes within the arsenal of electrochemists and make them a viable option for real-world applications, the development of better-quality filament was a prerequisite. We will now explore the efforts researchers have gone through to develop these bespoke, in-house filaments.

## 3 Bespoke Filament Design

To overcome the severe performance limitations and limited knowledge of compositions, in the past few years researchers have begun producing their own filament, allowing precise control over material compositions.^10^ The understanding of exact compositions has allowed researchers to develop deeper insights into the molecular level chemistry that affects the performance of additive manufactured electrodes and design enhanced systems for specific applications. The production of bespoke filament for additive manufacturing electrochemistry currently revolves around the combination of the base polymer, conductive or functional fillers, and plasticizer compounds, if required.

### 3.1 Base Polymer Materials

The base polymer used has a profound effect on the compositions available during filament production, what production methods can be used, and the characteristics of the final product. In particular, the choice of base polymer has significant implications for the loading of conductive filler possible, the method of incorporation of these fillers, and the mechanical and chemical properties of the final products. When considering FFF additive manufacturing as a whole, it is clear that PLA is the most commonly utilized filament, but we can also see a number of other thermoplastics used, such as acrylonitrile butadiene styrene (ABS), poly­(ethylene terephthalate glycol) (PETg), thermoplastic polyurethane/elastomers (TPU/TPE), polypropylene (PP), nylon and polycarbonate (PC), among others. Some of the structures and key properties of these polymers can be seen in Table 1.

**Table 1:**
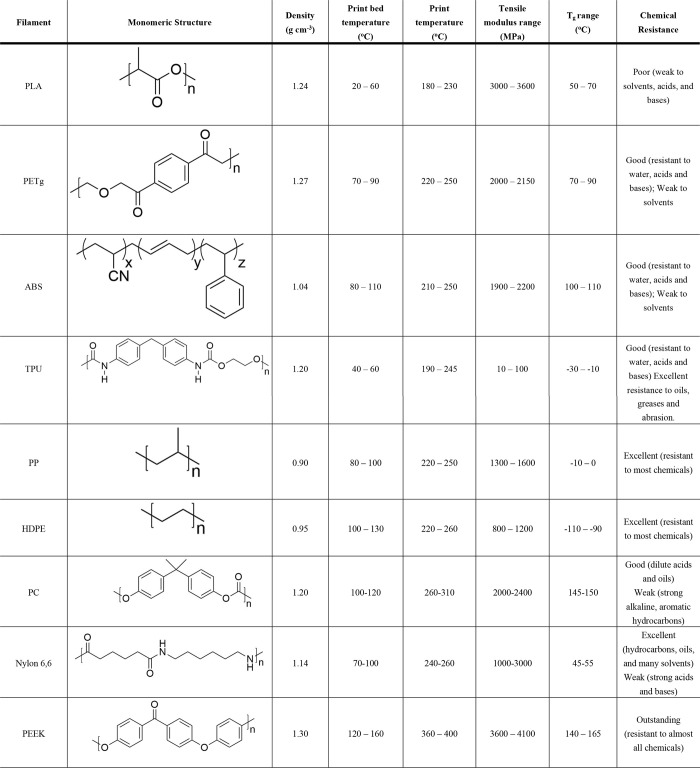
Chemical Structures and Some Key Physicochemical and Mechanical Parameters Associated with Thermoplastics Commonly Used in FFF^a^

a **Key:** PLA – poly­(lactic acid); PETg – poly­(ethylene terephthalate glycol); ABS – acrylonitrile butadiene styrene; TPU – thermoplastic polyurethane; PP – polypropylene; HDPE – high density poly­(ethylene); PC – polycarbonate; PEEK – polyether ether ketone.

If we first consider PLA, as the most synonymous polymer with FFF printing, it is widely adopted due to its ease of processing, dimensional stability, and cost-effectiveness.^150^ PLA is a thermoplastic aliphatic polyester derived from renewable resources such as corn starch or sugar cane, which contributes to its reputation as a sustainable material. However, while PLA is technically biodegradable under industrial composting conditions, such infrastructure is largely unavailable in most regions, meaning that its environmental benefits are often overstated.^151–153^ Physically, PLA exhibits a density of approximately 1.24 g/cm^3^, a glass transition temperature of 55–65 °C, and a melting range of 150–180 °C. Mechanically, it offers high stiffness with a tensile strength of 50–70 MPa and an elastic modulus near 3.5 GPa,^154,155^ though its impact resistance and thermal stability are limited, restricting its use in high-temperature or load-bearing applications. PLA’s popularity in FFF stems from its low printing temperature (190–220 °C), minimal warping, and ability to produce smooth surfaces with fine detail. Additionally, it emits negligible fumes during printing and is widely available at low cost, making it ideal for prototyping.^156^ Despite its widespread use in FFF, PLA is generally unsuitable for many applications due to its poor chemical stability^8^ and susceptibility to water ingress. The chemical stability of different filaments commonly used in additive manufacturing can be seen in Figure 7.

**Figure 7:**
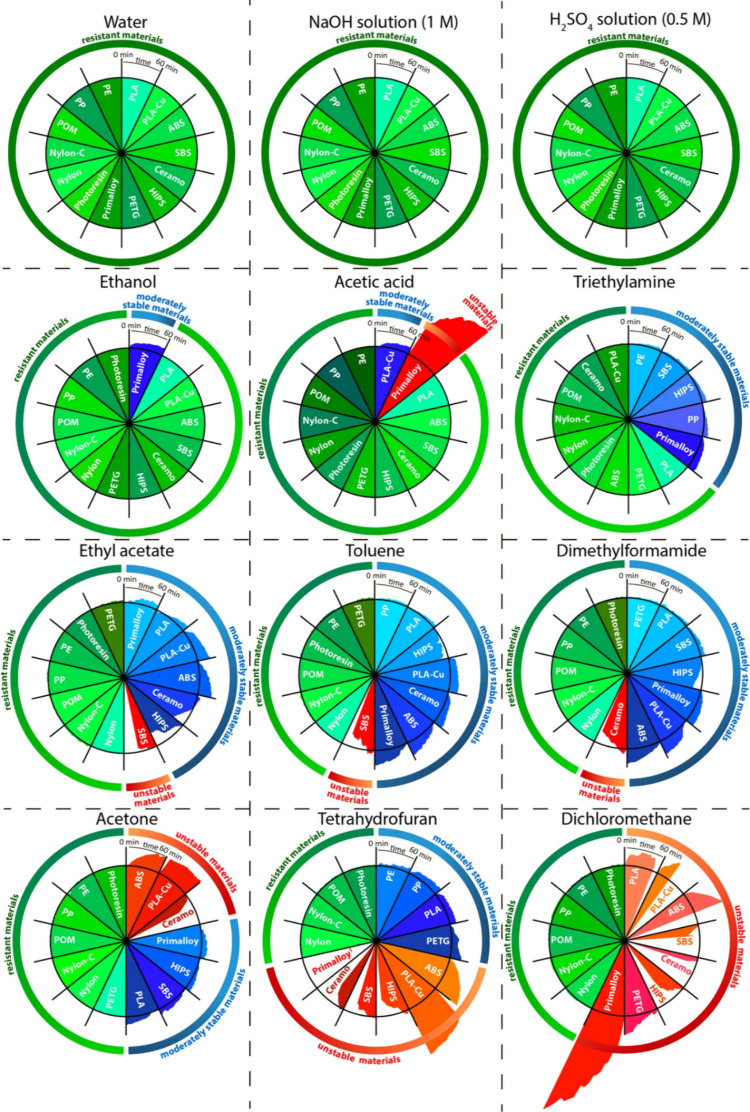
Figure showing the stability of different printed parts in various solvents. Reproduced from ref 8. Copyright Springer 2019.

ABS is a widely used thermoplastic in FFF due to its toughness, impact resistance, and relatively high heat stability compared to PLA. It has good dimensional stability when printed with a heated bed (90–110 °C) and extrusion temperatures of 220–250 °C. ABS offers moderate chemical resistance to aqueous solutions and dilute acids but is susceptible to degradation in strong oxidizing environments and organic solvents. Despite its mechanical robustness, ABS presents notable drawbacks in FFF, including the emission of potentially harmful fumes during printing, a tendency to warp without a heated bed, and sensitivity to environmental conditions such as humidity. PETg combines the ease of printing associated with PLA and the improved toughness of ABS. PETg is printed at 220–250 °C with a bed temperature of 70–90 °C and exhibits good resistance to water and many chemicals, including weak acids and bases. Although PETg offers good dimensional stability and chemical resistance, it can suffer from stringing and “oozing” during printing due to its low viscosity, and its tendency to absorb moisture requires careful filament drying to avoid surface defects and reduced mechanical performance. PP is valued for its excellent chemical resistance, low density and flexibility. It requires higher bed temperatures (90–110 °C) and extrusion temperatures of 220–250 °C to minimize warping during FFF. Its resistance to acids, bases, and many organic solvents makes it attractive for chemically aggressive environments. PP is challenging to print because of its high shrinkage and warping tendencies, which demand precise thermal control and often specialized build surfaces. Additionally, its low surface energy makes layer adhesion difficult, leading to poor interlayer bonding without optimized settings. TPU is a flexible, elastomeric material widely used in FFF for applications requiring impact absorption and elasticity. TPU prints at 210–240 °C with a bed temperature of 40–60 °C and offers excellent abrasion resistance and chemical stability against oils and many solvents. TPU’s flexibility makes it difficult to feed through standard extruders, often requiring direct-drive systems.^28^ It is prone to stringing and inconsistent extrusion due to its low stiffness, and slow print speeds are necessary to maintain dimensional accuracy. Additionally, TPU filaments are hygroscopic, so moisture absorption can lead to poor surface finish and reduced mechanical properties without proper drying.

Beyond the widely adopted commodity polymers used in FFF, a number of high-performance thermoplastics remain entirely unreported within additive manufacturing electrochemistry, yet offer substantial potential for future device development. These materials possess mechanical, thermal, and chemical properties that could enable applications not readily accessible with the current pool. HDPE is a chemically inert, low-cost polyolefin with excellent resistance to acids, bases, and organic solvents, making it promising for electrochemical systems that require prolonged exposure to harsh environments. Its low density and good toughness also make it suitable for lightweight, resilient device components. Although HDPE presents printing difficulties, including warping and poor interlayer adhesion due to its semicrystalline nature, recent advances in surface treatments, bed adhesion strategies, and controlled cooling have improved its printability. HDPE’s low surface energy challenges coating adhesion, but it also imparts exceptional chemical resistance, which is highly advantageous for long-term electrochemical stability. Conductive HDPE composites have been demonstrated in extrusion-molded or injection-molded formats, indicating that the development of a conductive HDPE FFF filament is feasible. Given its durability and recyclability, HDPE represents a particularly attractive candidate for environmentally sustainable electrochemical devices designed for field deployment.

PEEK is a high-performance engineering thermoplastic renowned for its exceptional chemical resistance, thermal stability, and mechanical strength. With a melting point around 343 °C and a glass transition near 143 °C, PEEK can withstand temperatures and chemical environments far beyond the capabilities of standard FFF filaments. This robustness makes it particularly attractive for electrochemical applications involving aggressive electrolytes, elevated temperatures, or high-pressure operation, such as flow cells, corrosion probes, or structurally demanding reactor components. PEEK’s stiffness and dimensional stability also lend themselves to precision-engineered electrochemical architectures. However, the absence of commercially available conductive PEEK filaments has so far prevented its adoption in electrochemical printing. The polymer’s compatibility with carbon-based fillers suggests that tailored conductive blends could be feasible, especially as high-temperature FFF systems continue to become more accessible. If printable conductive PEEK composites are developed, they could enable a new class of chemically resilient and long-lived electrochemical devices.

Nylon filaments offer a distinct combination of toughness, flexibility, and wear resistance, providing mechanical advantages not present in more brittle polymers such as PLA. These characteristics make Nylon a strong candidate for applications requiring durability and impact tolerance, including wearable housings, flexible sensor mounts, and mechanically compliant components integrated within electrochemical systems. Nylon’s intrinsic hydrophilicity may be advantageous for surface modification or metal deposition processes, potentially improving the adhesion of catalytic films or conductive coatings. However, the hygroscopic nature of polyamide introduces challenges for dimensional stability and electrochemical consistency, as water uptake can alter both mechanical and thermal properties. Although conductive Nylon composites exist for static-dissipative applications, none have yet been exploited for additive manufacturing electrochemistry. Developing moisture-resistant formulations or optimized drying protocols could unlock Nylon’s potential as a mechanically robust substrate for electrochemical devices.

PC is characterized by excellent impact strength, good thermal resistance, and partial optical transparency, qualities that position it uniquely for electrochemical applications where mechanical resilience or visual access to the reaction environment is desired. With a glass transition temperature around 147 °C, PC can tolerate higher operating temperatures than PLA or PETg, while its toughness supports the fabrication of pressure-resistant chambers, structural housings, and flow channels. The inherent transparency of PC opens opportunities for hybrid spectroelectrochemical systems, allowing simultaneous optical and electrochemical interrogation. While conductive PC composites have been demonstrated in other engineering fields, none have yet been adapted for FFF-based electrochemical device fabrication. As PC is compatible with carbon black, CNTs, and graphene fillers, future developments in conductive PC filaments could enable complex, multifunctional electrochemical architectures not achievable using currently available opaque materials.

The base polymers reported for use in electrochemical applications all bring their own unique properties to enhance performance. While the other polymers found in Table 1 are yet to be included reports for electrochemistry. We foresee this happening in the near future and note the ethos stated by Weyhrich and Long, in which they state “ . . . the additive manufacturing materials toolbox only contains a fraction of commercially available high-performance polymers due to unique processing constraints”.^157^ Considering the first truly conductive filaments utilizing TPU, PP and PETg have only been reported since 2024, see Figure 3, further expansion of the material pool is an inevitability.

### 3.2 Conductive and Functional Fillers

The inclusion of conductive fillers within the insulating polymer matrix is a prerequisite for the production of an electrically conductive filament. However, achieving a continuous conductive network within an otherwise insulating polymer requires a substantial filler loading, governed by the percolation threshold. The percolation threshold model describes the critical filler concentration at which an interconnected network of conductive particles forms throughout the polymer matrix, allowing electron transport. Below this threshold, conductive particles remain isolated, and the composite behaves as an insulator. Once the threshold is surpassed, conductivity increases dramatically, often following a power-law relationship with filler content. The exact threshold depends on factors such as filler morphology, aspect ratio, dispersion quality (significantly influence by production method, see later), and polymer-filler interactions.^158–160^ In addition to electrical conductivity, the intrinsic properties of the component materials directly affect the filament’s other material properties, including mechanical and thermal properties, as well as physical properties such as density.^10^ As the majority of filler materials are mechanically more robust than the polymer, many can reinforce the material if dispersed correctly throughout the matrix. On the other hand, agglomeration and nonuniform dispersion can act as stress-focusing regions, promoting material failure. This is a critical consideration when producing filament, even for electrochemical applications, as the filament must maintain integrity during the rapid printer movements during the printing process.

Within additive manufacturing electrochemistry the two main commercial conductive filaments, Black Magic and Protopasta, utilize graphene (Gn) and carbon black (CB) as their conductive fillers respectively. When considering the progression into bespoke filament production, carbon black stands out as the primary carbon-based conductive filler used. CB consists of fine, amorphous carbon particles, Figure 8A, with high surface area and intrinsic conductivity, which facilitates the formation of conductive pathways within a polymer matrix. Its popularity stems from its processability and economic advantages, with it being significantly cheaper to obtain than many of its counterparts such as Gn or multiwalled carbon nanotubes (MWCNT), while offering good conductivity (∼10^3^–10^5^ S cm^–1^).^10^ Considering the conductivity of other commonly used carbon fillers we see that Gn (10^6^–10^8^ S cm^–1^) and MWCNT (10^5^–10^7^ S cm^–1^) offer some of the best conductivities, with graphite (Gt) (10^4^–10^5^ S cm^–1^) offering similar to CB.^10^ Importantly, when creating conductive filament, it is not only the conductivity of the material that creates a good performing filament, as printability and flexibility are hugely important factors (often neglected in the literature). To understand why different fillers impact these key parameters in different ways we can look to their morphologies, Figure 8.

**Figure 8:**
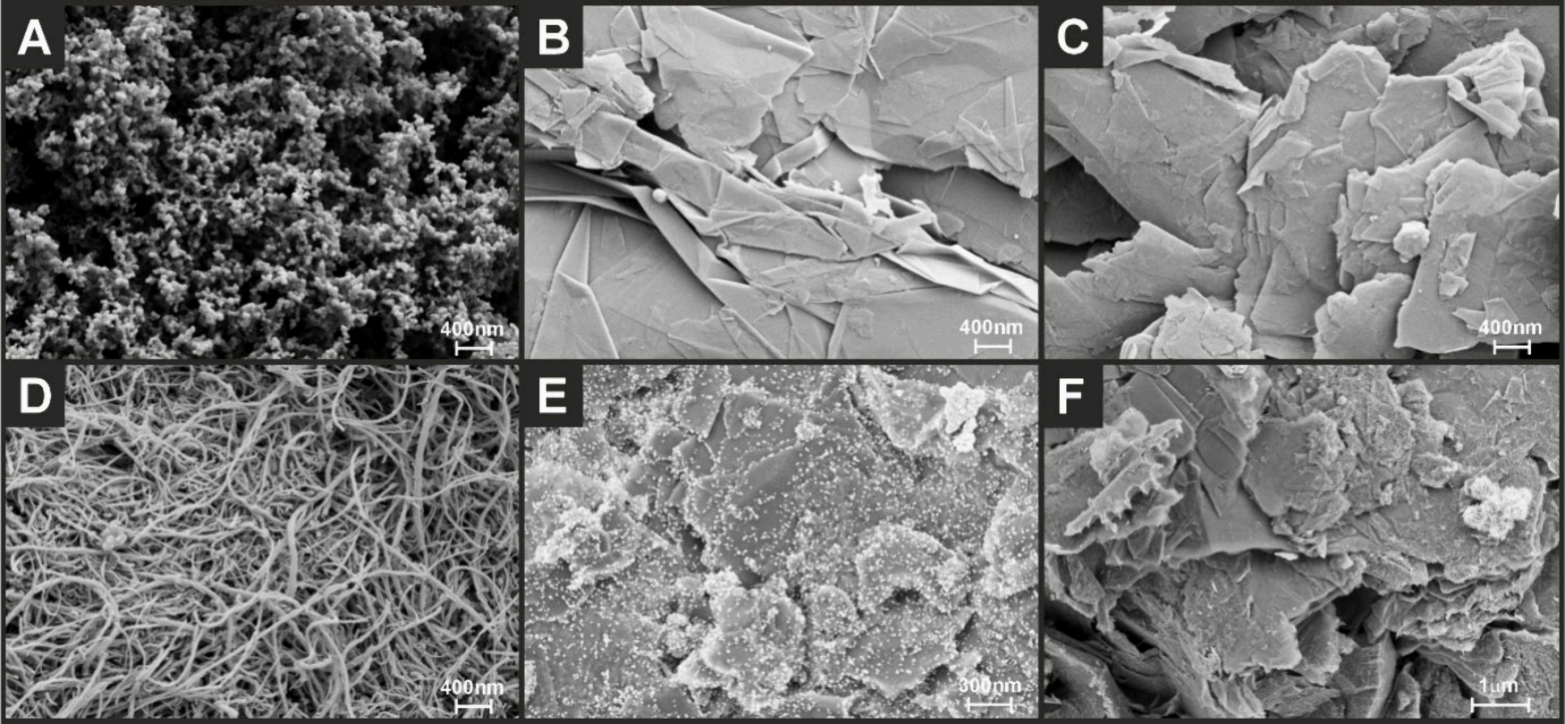
Scanning electrochemical micrographs of different powders used to produce conductive filament: (A) carbon black; (B) graphene; (C) graphite; (D) multiwalled carbon nanotubes; (E) PdNPs formed on graphite flakes; (F) FeNPs formed on graphite flakes.

Graphite, composed of layered platelets, offers moderate conductivity but its larger particle size and rigid morphology limit maximum loading without severely compromising printability and interlayer adhesion. In contrast, graphene sheets, with their two-dimensional morphology and extremely high aspect ratio, can achieve percolation at much lower loadings, potentially preserving flexibility and reducing viscosity increase; however, achieving uniform dispersion is challenging and often requires surface functionalization. Multiwalled carbon nanotubes (MWCNTs) provide the lowest percolation threshold due to their one-dimensional, tubular structure, forming efficient conductive networks at minimal concentrations. While this minimizes negative effects on flexibility, MWCNTs significantly increase melt viscosity even at low loadings, complicating extrusion and requiring precise control of processing parameters. Thus, filler morphology dictates a tradeoff between conductivity, mechanical integrity, and printability, with spherical fillers favoring ease of processing and high-aspect-ratio fillers enabling superior electrical performance at lower concentrations but demanding advanced dispersion techniques. One way in which researchers have looked to maximize both performance and printability of filaments, is through utilizing mixed morphologies, first reported for the combination of CB with graphite.^161^ In this way the authors ensured the maximum electrochemical performance was maintained, while significantly reducing the material cost of the filament, with their CB costing £0.26 per gram compared to £0.06 per gram of graphite. The combination of CB and Gt morphologies allowed for the maintenance of the conductive pathways through the filament, with a ratio of 3:2 in favor of CB found to be optimal within PLA,^162^ and 1:1 ratios being found within PP^163^ and TPU^164^ filaments. This tactic of mixed morphologies has been reported for CB and MWCNT to create a filament with further enhanced electrochemical performance in PLA,^165,166^ as well as three separate morphologies being used within PETg.^167^

On top of carbon-based fillers, there has been a recent trend (2024-onward, see Figure 3) of including functional fillers into the filament for additive manufacturing. This began with the inclusion of CuO and Bi_2_O_3_ by the group of Kokkinos.^85^ In this work, a solvent-based mixing step was used, whereby PLA, CB, the commercial functional powders, and poly­(ethylene glycol) (PEG) was dissolved and mixed within dichloromethane (DCM). Following dispersion, a film was cast to allow for the evaporation of the DCM, with the resultant filled polymer ground and then extruded to produce a filament suitable for additive manufacturing. These functional fillers improved the performance of the filaments toward heavy metal determination in the case of Bi_2_O_3_ and uric acid and glucose, in the case of CuO. The inclusion of Bi_2_O_3_ ^168^ and CuO^169^ have since been reported again for further uses. In 2025, there has been reports of the inclusion of precious metal nanoparticles (NPs) within conductive filament, summarized in Figure 7.

Through utilizing the reducing nature of graphite powder, the inclusion of Pt (Figure 9A),^89^ Au (Figure 9B),^88^ and Ag (Figure 9C)^87^ nanoparticles has been reported in PLA-based filament through a thermal mixing method, examples of SEM imagesfor Pd and Fe particles can be seen in Figure 8D and F. In each case, the NPs were formed directly on the surface of Gt flakes through mixing overnight within an aqueous solution, with no further chemicals required. The presence of the metal particles on the surface of the Gt were then confirmed through various physicochemical tests, including XPS, Figure 9A inset. The functionalized Gt was then used to make conductive filament in a preoptimized ratio of CB to Gt, alongside castor oil and recycled PLA (rPLA). Through thermally mixing the components at 190 °C for 5 min, and then extruding, the authors were able to produce a uniform filament, which improved the electrochemical performance in each case, even with a low loading of nanoparticles. One alternative strategy for the inclusion of AuNPs in particular has been shown by Bernalte et al.,^170^ who synthesized the AuNPs within castor oil, which was then used as a plasticizer in the formation of conductive filament alongside rPLA and CB. Although very low loading, significant improvements were seen in response to the detection of dopamine, Figure 9D. This unique strategy is viable for filaments where a plasticizer is incorporated, which we will now discuss.

**Figure 9:**
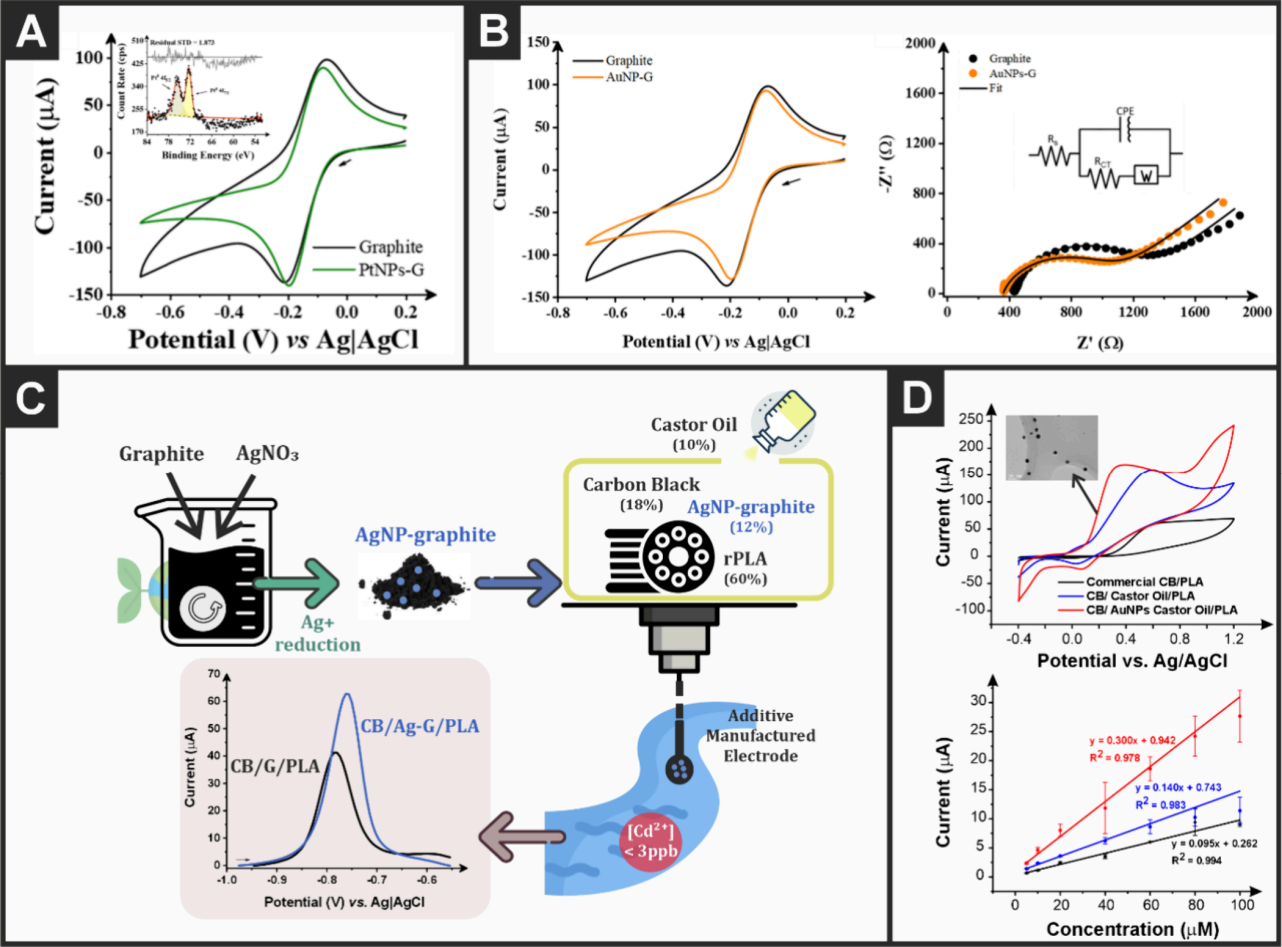
(A) Cyclic voltammograms comparing electrodes printed from a Gt-based filament and a Gt-PtNP filament, with inset XPS fit for Pt. Reproduced with permission from ref 89. Copyright Royal Society of Chemistry 2025, license CC-BY 3.0. (B) Cyclic voltammograms and Nyquist plots comparing electrodes printed from a Gt-based filament and a Gt-AuNP filament. Reproduced with permission from ref 88. Copyright Royal Society of Chemistry 2025, license CC-BY 3.0. (C) Schematic for the production of AgNP loaded filament and differential pulse voltammograms comparing the detection of Cd^2+^. Reproduced with permission from ref 87. Copyright Elsevier 2025, license CC-BY 4.0. (D) The detection of dopamine with AuNP loaded castor oil filament, with inset TEM image. Reproduced with permission from ref 170. Copyright Elsevier 2025, license CC-BY 4.0.

### 3.3 Plasticizers

Plasticizers are defined by the Council of the International Union of Pure and Applied Chemistry (IUPAC) as “a substance or material incorporated in a material (usually plastic or elastomer) to increase its flexibility, workability, or distensibility”.^171^ They help improve the quality of the filament, when high levels of filler are used, which is vital. Fragile or nonuniform filament can lead to breakages within various stages of the print cycle, including on insertion, in the extruder gears, between the extruder and hot-end, and during rapid printer movements. All of these instances result in failed prints, wasted material, and wasted time. Currently, it has been shown that conductive PETg, PP and TPU can be made at significantly high loadings, while still producing excellent flexibility, without the use of a plasticizer.^27,28,167^ While there are some reports of nonplasticized conductive PLA, in-particular using solvent-based production methods, the majority of currently reported bespoke and commercial filaments utilize a plasticizing compound, which allows the inclusion of much greater proportions of conductive filler and hence gives significant improvements in electrochemical performance. The plasticizer used within commercial Protopasta conductive PLA is proprietary information, for this reason molecular level understanding of the electrodes is near-impossible. However, for bespoke filaments significant numbers of materials have now been tested, beginning with PEG^143,172,173^ and poly­(ethylene succinate) (PES).^84^ Following this, there has been a movement toward biobased compounds as plasticizers, stemming from seminal work reporting castor oil (CO) at 10 wt% in a filament for the detection of bisphenol A.^82^ CO is an inedible oil that can be extracted through mechanical pressing or solvent extraction from the plant *Ricinus communis*, belonging to the Euphorbiaceous family. This plant is cultivated on industrial scales in multiple countries globally due to its many industrial uses, including India, China, Brazil, Thailand, Ethiopia, and the Philippines. Due to the fact it is inedible and can be grown globally on marginal lands, it has significant promise in the sustainable development of filaments. Other biobased compounds used as a plasticizer include Babassu oil^174^ and soybean oil, but these suffer from specific regional growing and deforestation issues, respectively.^175^ Recently a report comparing the electrochemical performance of filaments with 25 wt% CB loading, produced with 10 different plasticizing compounds (all set at 10 wt%) has been published.^116^ In this work they test PEG, PES, and CO, as well as dioctyl terephthalate, tributyl citrate, bis­(2-ethylhexyl) adipate, tris­(2-ethylhexyl) trimellitate (TOTM), diethylene glycol dibenzoate, diisodecyl phthalate (DIDP), and diisononyl phthalate (DINP). It was found that PEG can have a profound effect on the electrochemical performance, significantly increasing the capacitance of the systems, while CO, TOTM were identified as the other best performers.

### 3.4 Fabrication Strategies

Understanding the component parts of filament is crucial in their design and performance, but equally so is the method in which the filament is prepared. Within the literature there are two main methodologies reported, namely solvent mixing and thermal mixing (melt compounding). In both cases, these methods are only for producing the conductive composite through combining materials, both require and additional final extrusion step to produce the final filament ready for printing, summarized in Figure 10.

**Figure 10:**
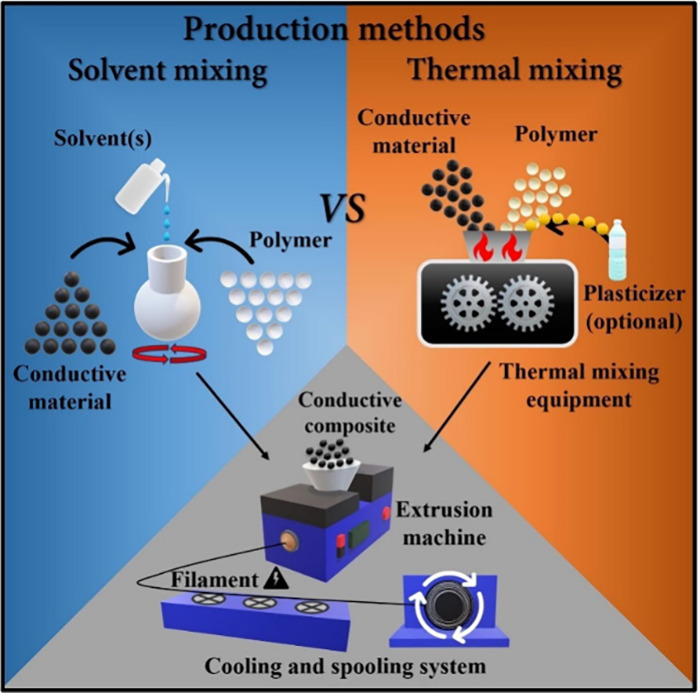
Schematic overview of the solvent and thermal mixing methods used for the production of conductive composites and filaments in FFF. Reproduced with permission from ref 10. Copyright Elsevier 2023, License CC-BY 4.0.

First, considering the solvent-based method, the materials chosen for filament fabrication and dissolved and/or dispersed within an appropriate solvent. There have been many reports of different solvents used toward this end; however, DCM, chloroform, and xylene are prominently found within the literature. The polymer granules are dissolved within the solvent matrix, which can be accelerated through heating, after which the filler components are added and mixed through simple stirring. Once the fillers are dispersed, the mixture is cast and then the solvent evaporated off, leaving the polymer composite ready to be reduced in size and then fed into an extruder. This method is popular due to the low start-up cost, as it can be performed using simple laboratory glassware. However, while simple and accessible, it suffers from long preparation times (days in some cases), low production volumes, and variable dispersion due to the low-shear method and difficulty in dispersing filler particles in nonenergetically compatible solvents.^10^

In comparison, the reported thermal mixing methods involves the addition of all component parts into a heated chamber, which is set depending on the base polymer used. Because of the high viscosity of polymer melts and the design of the mixer, high shears can be generated, which readily disperses the filler particles in minimal timeframes. We note that this should be verified within experimental reporting, as there are some reports emerging of thermal mixing methods on simple hotplates, which will not generate the same mixing performance.^175^ The majority of reports for thermal mixing involve only a 5 min mixing time to create the polymer composite, maximizing efficiency and minimizing degradation to the polymer through thermal exposure. An additional advantage of this system is the removal of all hazardous and toxic solvents; however this comes with a significant investment into production technologies not always available.

Although significant progress has been made in the development of bespoke conductive filaments for additive manufacturing electrochemistry, several fundamental issues remain unresolved and should be addressed to consolidate this emerging subfield. First, reproducibility in filament compounding is a key limitation. Variability in carbon allotrope dispersion, filler wetting, and polymer–filler interfacial compatibility can lead to batch-to-batch inconsistencies in conductivity, printability, and mechanical performance. Without standardized processing protocols and quantifiable quality control metrics, it remains difficult to compare results across laboratories or to predict device performance reliably. Second, material homogeneity along the filament length is still poorly understood and rarely characterized. Localized agglomeration, microvoid formation during extrusion, and fluctuations in filament diameter can introduce substantial spatial variation in electrochemical response. These inconsistencies ultimately undermine the fidelity of printed electrodes, particularly when fine geometries or low-surface-area designs are required. A third challenge lies in balancing filler loading with printability. While higher loadings improve bulk conductivity, they also increase viscosity, reduce melt flow, and can compromise layer adhesion, leading to mechanical brittleness or print failure.

## 4 Sustainability within Additive Manufacturing Electrochemistry

Sustainability has become a central consideration in materials science and manufacturing, driven by global efforts to mitigate environmental impact and promote resource efficiency.^176,177^ Within the context of FFF, sustainability encompasses reducing waste, minimizing energy consumption, and selecting materials with lower environmental footprints.^178^ Green chemistry principles, provide a foundation for this approach by advocating for the design of processes and products that reduce or eliminate hazardous substances, improve atom economy, and utilize renewable feedstocks wherever possible.^179–181^ The importance of sustainable development in additive manufacturing aligns with key international policy agendas. The United Nations Sustainable Development Goals (SDGs),^182^ particularly Goal 12 (Responsible Consumption and Production) and Goal 13 (Climate Action), emphasize the need for sustainable industrial practices. Similarly, the UK Industrial Decarbonisation Strategy^183^ and the EU Circular Economy Action Plan^184^ prioritize reducing carbon emissions, promoting material circularity, and advancing eco-innovation in manufacturing sectors. These frameworks underscore the role of additive manufacturing in achieving sustainability targets by enabling localized production, reducing material waste compared to subtractive methods, and supporting the transition to renewable and recyclable materials.

To this end, one of the key trends and advances made within additive manufacturing electrochemistry is the use of recycled polymers, rather than virgin feedstock. The first reports of this both utilize rPLA originating from postindustrial coffee pod waste (rPLA), where through combining it with CB and PEG or PES, filament suitable for the production of supercapcitors^173^ and sensors^84^ were made. In particular, the work from Sigley et al.^84^ signaled the introduction of Circular Economy concepts into additive manufacturing electrochemistry through several lenses, Figure 11A. First, nonconductive filament was produced from the rPLA and used to print the electroanalytical cells. Importantly, these cells were then recycled again after use, with the authors finding that the cells could be recycled back into filament and the cells reprinted with excellent integrity up to 4 times, creating a true closed cycle circular economy. For the electrodes, the rPLA (61.62 wt%) was combined with CB (29.60 wt%) and PES (8.78 wt%) to create a filament with good flexibility and printability, which was used to create working electrodes with significantly enhanced electrochemical performance compared to the Protopasta commercial benchmark. This improvement is emphasized in the electroanalytical application, where the bespoke electrodes with *no* postprint treatment (activation) significantly outperformed the activated commercial electrodes. The potential removal of postprint modification is an important step both in terms of sustainability and commercial uptake of this technology. The final application for this system was for the detection of caffeine within tea and coffee samples, creating another unique circular aspect to the work. The recycling of the components in this work (cells and electrodes) were made simple due to the individual printing and post assembly of the overall devices, allowing individual recycling strategies to be used for pure rPLA and composite PLA materials. This maximizes recycling, but means significant post print assembly and disassembly steps, which could influence commercial uptake. As such, concurrently, the same group looked at recycling mixed material cells.^83^

**Figure 11:**
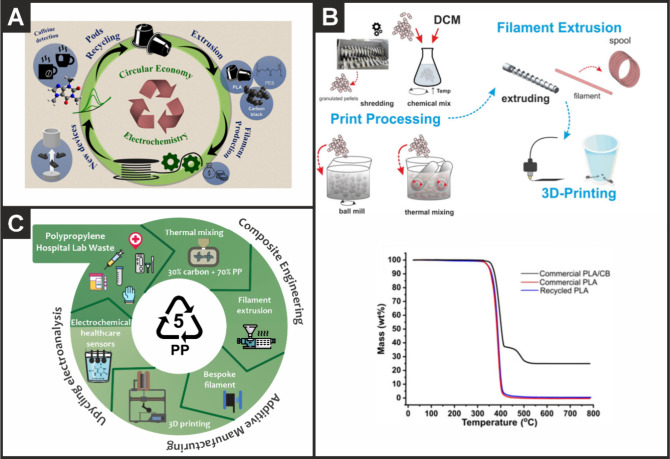
(A) Schematic representation of a circular economy electrochemistry, using postindustrial coffee pod PLA waste to create sensors for caffeine. Reproduced with permission from ref 84. Copyright American Chemical Society 2023, license CC-BY 4.0. (B) A summary of the strategies used to recycled mixed material prints back into filament for sensor production. Reproduced with permission from ref 83. Copyright American Chemical Society 2023, license CC-BY 4.0. (C) Schematic of the scheme used to upcycle hospital lab waste PP into conductive filament for sterilizable healthcare sensors. Reproduced with permission from ref 90. Copyright American Chemical Society 2025, license CC-BY 4.0.

This work considered electroanalytical platforms that are produced in a single print, using multimaterial or multiextruder systems, where the electrodes are effectively embedded into the wider cell. This allows full sensing platforms to be created in a single print, ready to use directly from the print bed, but also means that the conductive and nonconductive materials are fused together. They took a simple all-in-one cup cell design, previously published for the detection of acetaminophen^64^ and looked to recycle these cells through using four processing methodologies (granulation, ball-milling, solvent mixing, and thermal mixing), Figure 11B. The authors identified thermal mixing as the most effective approach, producing filaments with improved filler dispersion and mechanical integrity. Remarkably, the recycled nonconductive filament could be reused twice without supplementation from virgin polymer, while conductive filaments were created by incorporating carbon black during thermal mixing. These recycled filaments were then employed to fabricate a fully recycled electroanalytical cell, which retained the original design and achieved comparable performance for acetaminophen detection, with a sensitivity of 22.4 (± 0.2) μA μM^–1^, a limit of detection of 3.2 (± 0.8) μM, and recovery of 95 (± 5)% in real pharmaceutical samples. This work represents a paradigm shift toward circular economy electrochemistry, illustrating how waste sensors can be transformed into new sensing platforms without reliance on virgin feedstock.^83^

The use of recycled polymers for the creation of conductive filament has been extensively reported for PLA, but there have also been recent reports in the use of recycled PETg waste from additive manufacturing^86,167^ and PP waste from hospital laboratories,^90^ Figure 11C, to create conductive filament. Importantly, both of these filaments were capable of being sterilized without affecting the electrochemical performance, opening up potential uses within healthcare-based sensors and in the case of PP introducing another potential circular economy. Importantly, the upcycling work by Khan et al.,^90^ uses PP waste, which is one of the most utilized polymers worldwide in every day life. This work has the potential to not only divert significant volumes of PP from incineration or landfill but also leverage its inherent chemical resistance for robust sensor platforms. The resulting devices demonstrated reliable electrochemical performance for analyte detection, confirming that upcycled PP can serve as a viable alternative to virgin polymers in demanding applications. By transforming clinical plastic waste into high-value sensing systems, this work exemplifies how additive manufacturing can operationalize circular economy concepts within healthcare, aligning with UN SDG 12 (Responsible Consumption and Production) and SDG 3 (Good Health and Well-being) while reducing environmental burden.

Alternatively, there has been advancements in the sustainability of fillers used within filament production. First, there have been reports of replacing certain proportions of conductive filler with biochar.^91,185^ Biochar is a carbon-rich material derived from biomass pyrolysis and has gained attention as a sustainable precursor for electrochemical applications. Its inherent porosity, high surface area, and tunable surface chemistry make it an attractive candidate for electrode fabrication. Alternatively, a recent report has looked at the inclusion of microcellulose into a bespoke filament.^186^ In this work, the microcellulose was easily removed in postprinting, which made the conductive components significantly more available. This allowed the researchers to use a lower loading of conductive carbon while offering large improvements in the electrochemical performance.

Importantly, long-term stability, aging behavior, and environmental durability remain largely unexplored. Understanding how conductive filaments respond to humidity, repeated electrochemical cycling, UV exposure, and thermal fluctuations will be essential for transitioning from laboratory demonstrations to deployable devices. This is especially important for applications in environmental sensing, wearable electronics, and energy systems where mechanical fatigue and chemical degradation are inevitable over extended use.

## 5 Applications for Electrochemical Devices

Additive manufacturing has emerged as a transformative platform for electrochemical applications, enabling innovative device architectures and material combinations that were previously impractical with conventional fabrication methods. The versatility of FFF and related techniques has driven research across diverse domains, including energy storage, hydrogen generation, and chemical sensing, where the ability to produce bespoke, integrated, and cost-effective systems is highly advantageous. These applications leverage the design freedom of additive manufacturing to optimize electrode geometry, enhance mass transport, and incorporate functional materials directly into printed structures. In the following sections, we explore the breadth of this literature, beginning with energy storage technologies, such as batteries and supercapacitors, where additive manufacturing has been employed to improve performance, reduce material waste, and enable novel configurations that align with the growing demand for sustainable and efficient energy solutions.

### 5.1 Energy Storage

Table 2 provides an overview of the state of literature on additive manufacturing for both batteries and supercapacitors. When analyzing this, we can see two clear trends, in which either commercially available filament is modified post print with materials, or these materials are integrated into the filament directly before printing. For additive manufacturing to deliver high-performance electrochemical energy storage devices, several key characteristics must be optimized. First, maximizing the surface area of electrodes is critical for enhancing charge transfer and ion accessibility, which directly impacts energy and power density. Equally important is the active material loading, as higher concentrations of electrochemically functional components increase capacity but often challenge printability and mechanical integrity. Electrical conductivity must also be carefully engineered, typically through the incorporation of conductive fillers or postprocessing treatments, to ensure efficient electron transport across the printed structure. Finally, achieving synergy between active materials and the polymer matrix is essential, not only to maintain structural cohesion during printing but also to preserve electrochemical performance without compromising flexibility or dimensional stability. Balancing these factors underpins the design of advanced additive manufacturing strategies for batteries, supercapacitors, and other energy storage systems, where geometry, material composition, and processing conditions converge to define device functionality.

**Table 2:** Summary of the Literature for Batteries and Supercapacitors Using FFF Additive Manufacturing^a^

Filament	Activation	Modification	Electrolyte	Specific Capacity	Energy Density	Stability	ref
Black Magic	–	–	1 M LiPF_6_	40 mAh g^–1^	–	∼85%	^72^
PLA/CB/Gt/MWCNT/LiFePO_4_ and Black Magic	–	–	1 M LiPF_6_	80 mAh g^–1^	–	–	^187^
PLA/Gt	–	–	1 M LiPF_6_	200 mAh g^–1^			^188^
ABS/PVA/CB/NaMnO_2_	Sonication in water	–	EMIBF_4_	84.3 mAh g^–1^			^73^
PLA/Gn	Chemical (1 M NaOH)	–	1 M LiPF_6_	500 mAh g^–1^			^189^
Black Magic	Chemical (1 M NaOH)	Electrodeposition Ti_3_C_2_@PPy	1 M H_2_SO_4_	–	2.64 Wh kg^–1^	81.4%	^190^
Protopasta	Chemical (DMF)	Electrodeposition PoPD	LiMn_2_O_4_	69.1 mAh g^–1^		84.4%	^191^
Black Magic	Chemical (DMF)	Electrodeposition MoS_x_	1 M H_2_SO_4_		0.2 μW h cm^–2^	∼90%	^192^
PLA/CB@PPy/Si/WJM-FLG	–	–	1 M LiPF_6_	345 mAh g^–1^		96%	^193^
PLA/MWCNT/AC/MoS_2_/PEG	Chemical (DMF)	–	1 M H_2_SO_4_			92%	^172^
Black Magic	Thermal (110 °C)	Electrodeposition	2 M ZnSO_4_	214.6 mAh g^–1^		78.1%	^194^
		PANI-PAA					
Black Magic	Chemical (DMF)	ALD V_2_O_5_	2 M ZnSO_4_	425 mAh g^–1^		78.5%	^195^
PLA/PEGDE/MWCNT/CB/LFP	Thermal (600 °C)	–	1 M LiPF_6_	154.2 mAh g^–1^	233.69 Wh kg^–1^	69%	^196^
Protopasta	Thermal (350 °C)	Electrodeposition	2 M KOH		4.74 mW cm^–2^	93%	^197^
		NiCo-LDH					
PLA/Ti_3_AlC_2_/PEG	Electrochemical (4 M NaOH)	Chronoamperometry (9 M HCl @ 5 V)	1 M LiCl	–	–	–	^198^
TPU/LFP/KB			1 M LiPF_6_	129.8 mAh g^–1^		∼98.5%	^199^
PLA/CB/PEG	Electrochemical (0.5 M NaOH)	–	17 M NaClO_4_		433.32 μW cm^–2^	92.86%	^173^
PLA/Ag/rGO@PANI-DBSA	–	–	1 M H_2_SO_4_		7 Wh kg^–1^	91%	^200^
Black Magic	Chemical (4 M NaOH)	Anode: PANI	1 M Li_2_SO_4_		5.10 μW cm^–2^	74%	^201^
	Electrochemical (2 V in PBS)	Cathode: Ti_3_C_2_T_x_					
PLA/Al_2_O_3_-rGO@PANI-DBSA			1 M H_2_SO_4_		15 Wh kg^–1^	85%	^202^
Protopasta	Chemical (6 M KOH)	–	6 M KOH		0.18 Wh kg^–1^	30%	^104^
PLA/CB/rGO/PB-Nb_2_O_5_	5 Stabilization cycles		1 M KCl	17.52 mAh g^–1^		85–92%	^203^
PLA/CNF	Anode:Chemical (4 M NaOH)	Cathode: Electrodeposited Zn	2 M ZnSO_4_	195 mAh g^–1^		78.32%	^204^
Black Magic	MWCNT		6 M KOH		11 Wh kg^–1^	96%	^205^
PLA/MWCNT/CB/Gt			1 M LiPF_6_	1.69 mAh cm^–2^			^206^
PLA/Gt/Ni-PB	Mechanical		1 M KNO_3_			73.7%	^207^
	Electrochemical (1 mM KCl)						
Black Magic	Chemical (4 M NaOH)	Bi_2_Te_3_	1 M (NH_4_)_2_SO_4_		134.8 Wh kg^–1^		^208^

a **Key:** CB – carbon black; Gt – graphite; MWCNT – multiwalled carbon nanotubes; PLA – poly­(lactic acid); ABS – acrylonitrile butadiene styrene; PVA – poly­(vinyl alcohol); EMIBF_4_ – 1-ethyl-3-methylimidazolium-bis-tetrafluoroborate; Gn – graphene; PPy – poly­(pyrrole); PoPD – poly­(ortho-phenylenediamine); DMF – dimethyl formamide; WJM-FLG – wet-jet milling-exfoliated few-layers graphene; AC – activated charcoal; PEG – poly­(ethylene glycol); PANI – poly­(aniline); PAA – poly­(acrylic acid); ALD – atomic layer deposition; LFP – lithium iron phosphate; PEGDE – poly­(ethylene glycol diglycidyl ether); LDH – layered double hydroxide; KB – Ketjen black; rGO – reduced graphene oxide; DBSA – dodecylbenzene sulfonic acid; PB – Prussian blue; CNF – carbon nanofibers.

To this end, Park et al.^196^ have reported the production of a PLA filament, with an ultrahigh loading of active materials (65 wt%) far exceeding typical AM battery feedstocks, by carefully balancing polymer binder content with conductive additives to maintain printability. Figure 12A shows images of the produced filament, here through exploring different loadings (0, 5, 10 wt%) of poly­(ethylene glycol diglycidyl ether) (PEGDE) plasticizer alongside 65 wt% active material, CB and MWCNT. They state the importance of plasticizer loading to printability of the filaments, with no plasticizer the filament was too brittle to withstand rapid printer movements or the pressure from the extruder gears, leading to filament fracture. In contrast, too much plasticizer led to a filament that was too flexible for the printing system they were using. It has been shown in other work printing highly flexible conductive materials that the printability of these filaments is highly dependent on the print head set up, with the type of extrusion system and the distance between the extruder and hot-end playing pivotal roles.^28^ These filaments were used to fabricate interdigitated electrode architectures via FFF, leveraging design freedom to enhance ion transport and reduce tortuosity compared to conventional slurry-cast electrodes. Postprint carbonization further improved electrical conductivity and mechanical strength, enabling the printed electrodes to function as load-bearing components while delivering an a_real_ capacity of 12.28 mAh cm^–2^.

**Figure 12:**
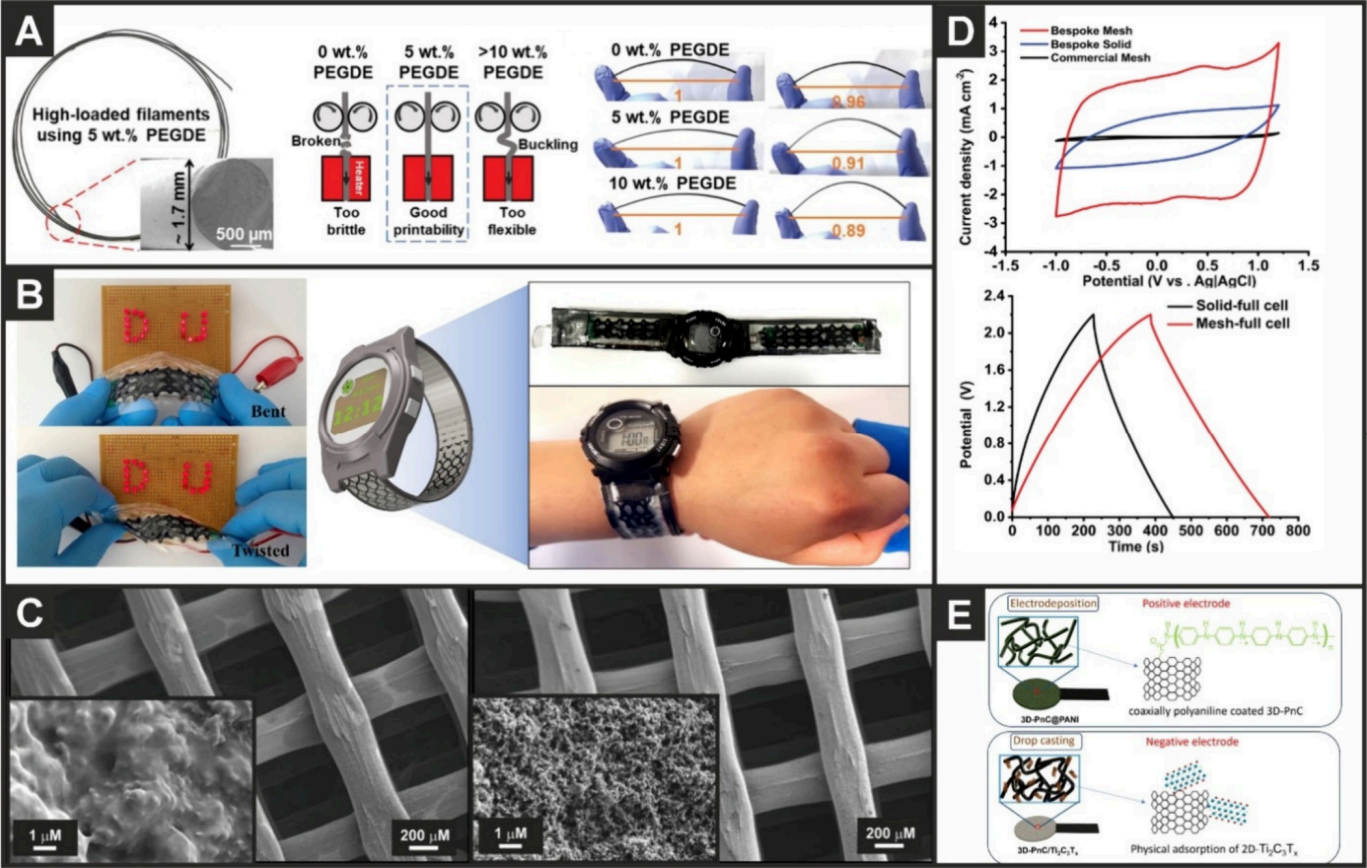
(A) A picture of the highly loaded cathode filament using 5 wt% PEGDE, a schematic showing the printability of the cathode filament based on plasticizer content, and a picture of the flexibility of the filament. Reproduced with permission from ref 196. Copyright Wiley 2023. (B) Photographs of the printed TPU based conductive material and its application in a watch strap. Reproduced with permission from ref 199. Copyright Wiley 2024, license CC-BY 4.0. (C) SEM micrographs of the lattice structure produced with a rPLA/PEG/CB filament. (D) Performance of the solid and mesh electrodes printed from the rPLA/PEG/CB filament. Both reproduced with permission from ref 173. Copyright Wiley 2023, license CC-BY 4.0. (E) Schematic for the deposition of MXene onto an electrode printed from commercial filament. Reproduced with permission from ref 201. Copyright Taylor & Francis, license CC-BY 4.0.

Through tailoring the base polymer and composition of bespoke filaments, additive manufacturing has the potential to truly revolutionize wearable energy storage devices. In this way Hu et al.^199^ have introduced a breakthrough in flexible energy storage by combining topology optimization with additive manufacturing to produce electrodes that exhibit both high electrochemical performance and exceptional mechanical resilience. The authors fabricated topologically structured electrodes (TSEs), Figure 12B, designed to minimize stress concentrations and maintain integrity under extreme deformation. The composite was produced through a solvent method, whereby TPU (55 wt%) was dissolved in DMF followed by the addition of lithium iron phosphate (39.5 wt%) as the active material and Ketjen Black (5.5 wt%) as the conductive component. After mixing and solvent evaporation the composite was processed though a single-screw extruder to produce the desired filament. It is important to note the slow printing speed (15 mm s^–1^) used in this work, which is common when using highly flexible materials such as TPU. Finite element analysis and mechanical testing confirmed that TSEs outperform conventional mesh-structured electrodes, sustaining up to 50% stretch without cracking and retaining 98% of their original capacity after 50 stretching cycles. This contrasts sharply with traditional designs, which suffer significant capacity loss and structural failure under similar conditions. The optimized architecture not only enhances flexibility but also preserves lithium-ion battery performance, making these electrodes ideal for next-generation wearable electronics and biointegrated devices.

Unique electrode designs and mesh structures in-particular are a real advantage of additive manufacturing for the creation of electrodes, as the toolpath can be precisely controlled, especially through the use of novel software such as FullControl GCode Designer.^209^ One of the first reports to compare solid and mesh electrodes using FFF additive manufacturing was achieved through combining the above software with a bespoke rPLA (67.5 v/v%) filament, made through thermal mixing with CB (22.5 v/v%) and PEG (10 v/v%), Figure 12C.^173^ By programming print paths and infill patterns directly through G-code, the study achieved tailored porosity and surface area without additional postprocessing, demonstrating how additive manufacturing can integrate structural and electrochemical functionality in a single manufacturing step. Figure 12D shows the enhancement in performance obtained from the mesh compared to solid electrodes, where the additionally interlayer spacing contributes to a 3.5 times improvement in electrode performance. This was attributed to the increase in available active surface area, ion accommodating capabilities, and faster ion diffusion.

All of the above use the production of bespoke filament to incorporate active/conductive materials, but there is still room for the use of commercial filament in the exploration of materials. In work from Mappoli et al.^201^ postprint surface modification was utilized, where the printed electrodes were sequentially coated with MXene and polyaniline (PANI) to create a synergistic composite structure, Figure 12E. MXene provides high electrical conductivity and rapid ion transport, while PANI contributes pseudocapacitive behavior, collectively enhancing energy storage performance. However, it is important to note that due to the resistance added into the systems from the commercial conductive filament, the works must utilize the design and production freedoms provided by additive manufacturing otherwise the exploration of materials should be completed using classical electrodes such as glassy carbons, that won’t introduce additional limitations.

### 5.2 Energy Conversion

Additive manufacturing offers unique advantages for electrodes used in hydrogen generation. Hydrogen evolution and oxygen evolution electrodes demand characteristics that optimize catalytic activity, mass transport, and gas release. High surface area and controlled porosity are critical to facilitate efficient reactant access and bubble detachment, while robust electrical conductivity ensures minimal ohmic losses during high-current operation. Additive manufacturing enables precise control over electrode geometry, such as lattice structures, graded porosity, and flow channels, through direct G-code programming, which cannot be easily achieved with conventional manufacturing. Furthermore, these electrodes often require chemical stability in highly alkaline or acidic environments, distinguishing them from energy storage electrodes that prioritize intercalation and mechanical flexibility. By leveraging design freedom and material versatility, additive manufacturing provides a pathway to tailor electrode architecture for enhanced catalytic performance, durability, and scalability in hydrogen generation systems.

Table 3 provides an overview of the literature reports on hydrogen generation using FFF additive manufacturing. Similarly to the section above, there are strategies that utilize commercial conductive filament, coating it postprint with catalytic material, or the incorporation of catalytic material into the filament. One of the first reports that exemplifies how additive manufacturing can be fully exploited to revolutionize electrode design for hydrogen generation was from Bui and co-workers.^210^ The authors developed a membraneless water electrolysis system using additive manufactured electrodes with intricate, interdigitated geometries that maximize surface area and minimize ionic resistance without relying on conventional separators, Figure 13A. The design incorporates tailored flow channels and optimizes spacing to enhance bubble detachment and mass transport, addressing limitations of planar electrodes in gas evolution reactions. This level of architectural precision, achievable only through additive manufacturing, enables improved efficiency and compactness, reducing material usage. Issues remain with this set up, primarily from the commercial filament used, which introduces significant resistance even after Ni electroplating on the surface of the electrode. The long-term stability of systems like this, especially within alkaline electrolyzers is an issue, as we saw in the activation section strong base solutions are often used to remove PLA from printed structures, which upon prolonged exposure will case part failure. Recent and further developments in the expansion of material feedstock for additive manufacturing electrochemistry makes this work prominent again. Even so, the study demonstrates how additive manufacturing can move beyond simple replication of traditional designs to create entirely new electrode architectures that integrate structural, fluidic, and electrochemical functionality, marking a significant advance in hydrogen generation technology.

**Table 3:** Table Summarizing the Publications for Energy Conversion Applications Using Fused Filament Fabrication^a^

Filament	Activation	Modification	Electrolyte	Overpotential	Tafel Slope (mV dec^–1^)	Stability	ref
Protopasta		Electrodeposition	1 M NaOH	–0.6 V @ 50 mA cm^–2^	105	70 mV increase over 4 h	^210^
		Ni					
PLA/Pt/C			0.5 M H_2_SO_4_	∼−0.6 V @ 10 mA cm^–2^	43	10 out of 1000 CV stable	^211^
Black Magic	Thermal (350 °C)	Electrodeposited	0.5 M H_2_SO_4_	∼−0.3 V @ 10 mA cm^–2^	119	–	^212^
		MoS_x_					
Black Magic	Thermal (350 °C)		0.5 M H_2_SO_4_	–0.85 V @ 10 mA cm^–2^	–	–	^76^
Black Magic	Chemical (DMF)	Dipcoat MXene	0.5 M H_2_SO_4_	–	316	–	^213^
		Dipcoat MoSe_2_			260		
Protopasta	Chemical (Ethanol)	Electrodeposited	1 M KOH	–	–	–	^214^
		Ni_x_Cu_x_					
PLA/Cu	Thermal (1075 °C)	Dipcoat Pd^2+^	0.5 M H_2_SO_4_	–0.44 V @ 10 mA cm^–2^	188	2 h stability @ 10 mA cm^–2^	^215^
	Chemical (1 M HNO_3_)						
Black Magic	Chemical (Ethanol)	Electrodeposited	1 M KOH	–0.27 V @ 10 mA cm^–2^	139	–	^216^
		NiPt_x_					
PLA/Gn/CNF	Chemical (KOH)		1 M KOH	–	–	–	^217^
Black Magic		Electrodeposited	1 M KOH	–0.102 V @ 10 mA cm^–2^	165	20 h tests	^218^
		NiCo_x_					
Black Magic	Chemical (DMF)		1 M H_2_SO_4_	–1.05 V @ 1 mA cm^–2^	–	–	^219^
			1 M KOH	–0.34 V @ 10 mA cm^–2^			^220^
PLA/Conductive paint		Electrodeposited	1 M KOH	–0.34 V @ 10 mA cm^–2^	–	–	^221^
		Ni-Mo					
PLA/MWCNT-Cu/B	Electrochemical (KOH)	–	1 M KOH	–0.29 V @ 1 mA cm^–2^	73	30 min tests	^222^
Black Magic	Chemical (NaOH)	ALD (Al_2_O_3_)	0.5 M H_2_SO_4_	–0.31 V @ 10 mA cm^–2^	–	–	^223^
Protopasta		Electrodeposited Ni-Fe	1 M KOH	–0.41 @ 10 mA cm^–2^	–	1 h tests	^224^
Inconel-625	Thermal (∼1200 °C)	–	0.5 M H_2_SO_4_	–0.52 V @ 20 mA cm^–2^	76	80 h	^225^
PLA/CO/CB/Gt-Pt	Mechanical	–	0.5 M H_2_SO_4_	–0.31 V @ 10 mA cm^–2^	46	7 h	^89^

a **Key**: PLA – poly­(lactic acid); DMF – dimethyl formamide; Gn – graphene; CNF – carbon nanofiber; MWCNT – multiwalled carbon nanotubes; ALD – atomic layer deposition; CO – castor oil; CB – carbon black; Gt – graphite.

**Figure 13:**
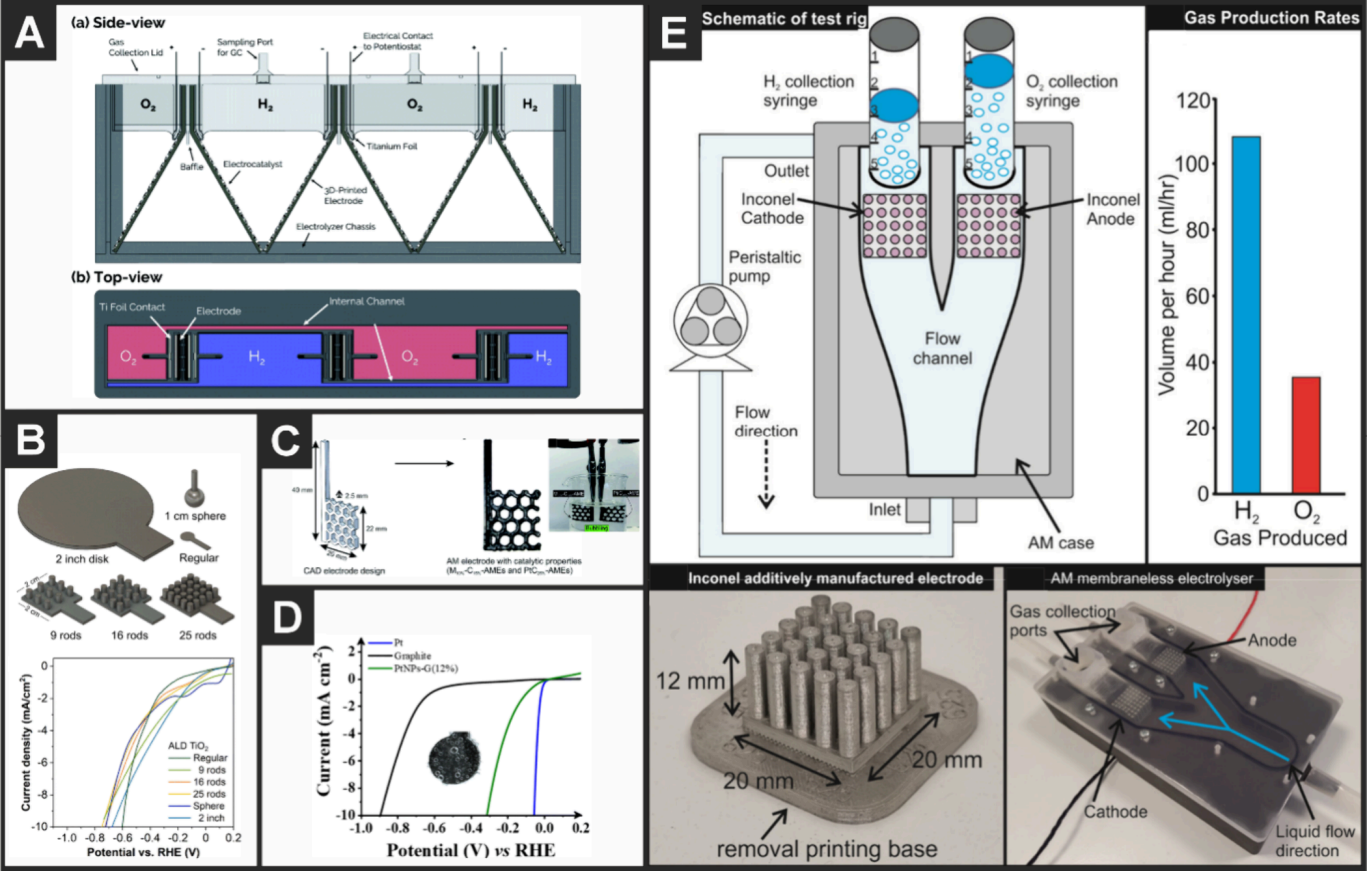
(A) Schematic of the modular membraneless electrolyzer with additive manufactured “clickable” electrodes. Reproduced with permission from ref 210. Copyright Royal Society of Chemistry 2020. (B) Schematic and LSV performance of electrodes with different rod morphologies. Reproduced with permission from ref 222. Copyright Royal Society of Chemistry 2024. (C) Flag designed electrodes from bespoke filament. Reproduced with permission from ref 211. Copyright Royal Society of Chemistry 2020, license CC-BY 3.0. (D) LSVs and inset additive manufactured electrode made from bespoke rPLA/Gt-PtNP filament showing the evolution of hydrogen gas bubbles. Reproduced with permission from ref 89. Copyright Royal Society of Chemistry 2025, license CC-BY 3.0. (E) Schematic design, gas production rates, and photographs of the electrodes and electrolyzer for an AM membraneless electrolyzer produced with Inconel-625 electrodes. Reproduced with permission from ref 225. Copyright American Chemical Society 2025, license CC-BY 4.0.

Other examples of using the design freedoms from additive manufacturing for hydrogen generation can be seen in Figure 13 B and C. In the first example, electrodes are printed with different geometric structures from commercial filament, and then coated with catalysts to aid performance through atomic layer deposition.^222^ Conversely, the work from Hughes et al.^211^ created bespoke filaments, incorporating Pt/C catalyst (25 wt%) in a PLA based filament. The electrodes produced from this filament produced improved hydrogen evolution compared to similar filaments produced with MoS_2_ and MoSe_2_. The authors benchmarked this filament and then utilized it to create the novel flag electrodes seen in Figure 13C, increasing the viable surface area. However, the performance was still significantly substandard due to the ohmic loss introduced into the system from the filament. Improvements in this regard have been seen by work from Augusto et al.,^89^ where an onset potential of −0.04 V decade^–1^ and overpotential at 10 mA cm^–2^ of −0.31 V is reported, compared to −0.09 V and ∼ −0.6 V for the previous work. This work uses the eco-friendly synthesis mentioned earlier, whereby potassium hexachloroplatinate­(IV) and graphite flakes are immersed in an aqueous solution overnight to synthesize the PtNPs on the surface of the graphite. The PtNP-Gt is then incorporated into the rPLA based filament through thermal mixing with CO and CB. Importantly, this uses the optimized graphite loading within PLA filament,^162^ meaning the ohmic loss is reduced. This work signals the importance of balancing catalyst incorporation and filament conductivity for hydrogen generation using additive manufactured electrodes.

A final example of the uses of additive manufacturing electrochemistry is seen in Figure 13E, which utilizes a Markforged Metal X 3D-printer.^225^ Building on earlier work in membraneless water electrolysis, this study advances durability and performance through a multitechnique additive manufacturing approach. The authors fabricated a robust electrolyzer using Inconel-based filaments, selected for their exceptional corrosion resistance in harsh electrochemical environments. Unlike previous designs relying solely on polymer-based FFF, this system integrates multiple AM processes to produce complex, high-strength components capable of withstanding prolonged operation under aggressive conditions. The architecture retains the benefits of membraneless design, simplified assembly and reduced cost, while leveraging the full potential of additive manufacturing for geometric optimization, including tailored flow channels and electrode positioning to enhance gas evolution and mass transport. By combining material innovation with design freedom, this work demonstrates how additive manufacturing can deliver electrolyzers that are not only efficient but also mechanically and chemically resilient, marking a significant step toward scalable hydrogen generation technologies.

### 5.3 Electrochemical Sensors

The extent of the literature for used of additive manufacturing electrochemistry for the production of sensing devices is vast in comparison to the other areas, summarized extensively in Table 4, where importantly the filament used, treatment methods and the analyte targeted are all included. From inspection of Table 4 we can observe a few key trends within the field, that can group research together. In the early stages of research into additive manufacturing electrochemistry for sensors much of the work was performed on commercial filament and looking different ways to improve the performance through postprocessing. In these works many classical analytes were targeted such as dopamine, glucose, Pb^2+^, acetaminophen and ascorbic acid, which offer a good way to benchmark the electrode performances. From this point research using commercial filament has progressed in three main ways. First, through the application of basic printed electrodes to different analytes. Second, to modifying the surface of basic electrodes, for example through dropcasting or electropolymerisation. Neither of these tactics really necessitate the use of an additive manufactured electrode and could be studied on any electrode. The third route of research using commercial filament focuses on the development of novel electrodes or full electrochemical cells that are only achievable in a laboratory environment due to the rapid prototyping and processing of additive manufacturing. As such, even though commercial filament is not at a standard to truly be viable for commercial sensing platforms, these works still offer significant progression to the field as these designs can be taken forward in future works. To this end, Figure 14A shows one of the first reported fully additively manufactured electroanalytical set up.

**Table 4:** Table Summarizing the Publications for Electroanalytical Applications Using Fused Filament Fabrication^a^

Filament	Activation	Modification	Analyte	Linear Range	Limit of Detection	Real Sample	ref
Protopasta	Electrochemical (NaOH)	–	Dopamine	1–250 μM	0.1 μM	–	^74^
Protopasta	–	–	Caffeine	0–90 mg L^–1^	1.8 mg L^–1^	–	^226^
			Hg^2+^	0–80 μg L^–1^	1.9 μg L^–1^	–	
			Glucose	2–28 mM	–	–	
Black Magic	Electrochemical (PBS)	–	Dopamine	2–10 μM	0.24 μM	–	^106^
Black Magic	Thermal (350 °C)		Picric acid	5–50 ppm	0.1 ppm	–	^101^
Black Magic	Digestion (Proteinase K)	Alkaline phosphatase	1-naphthol	–	–	–	^227^
Black Magic	–	–	Catechol	1–500 μM	1.4 μM	Artificial cerebral spinal fluid	^228^
Black Magic	Mechanical	–	TNT	1–870 μM	0.4 μM	Contaminated surfaces	^229^
PLA/PCL/ SBS/MWCNT	–	–	H_2_O_2_, NADH	–	–	–	^230^
Protopasta	Mechanical and Electrochemical (NaOH)	–	Pb^2+^	10–870 μg L^–1^	0.8 μg L^–1^	Tap water, liquid fuel ethanol	^231^
			Cu^2+^	10–870 μg L^–1^	2.0 μg L^–1^		
Black Magic/ Ni(OH)_2_	Mechanical and Electrochemical (NaOH)	–	Glucose	75–1000 μM	2.4 μM	–	^232^
Protopasta	Mechanical and Electrochemical (NaOH)	–	TNT	5–500 μM	1.5 μM	–	^233^
Protopasta	Mechanical and Electrochemical (NaOH)	–	Cd^2+^	30–270 μg L^–1^	2.9 μg L^–1^	Urine, saliva	^109^
			Pb^2+^	30–270 μg L^–1^	2.6 μg L^–1^		
Black Magic	Chemical (NaOH)	–	Dopamine	5–100 μM	1.67 μM	Synthetic urine, human serum	^12^
				100–1500 μM			
Black Magic	Chemical (DMF) Electrochemical (PBS)	–	Zearalenone	10–300 μM	0.34 μM	–	^234^
Black Magic	Chemical (DMF, HNO_3_, NaBH_4_)	–	Serotonin	0.3–10 μM	0.032 μM	Synthetic urine	^235^
PLA/Gt	–	–	Pb^2+^	0.19–4.5 mg L^–1^	0.16 mg L^–1^	–	^75^
			Cd^2+^	0.79–4.5 mg L^–1^	0.32 mg L^–1^		
Protopasta	Mechanical and Electrochemical (NaOH)	–	Ascorbic acid	5–20 μM	0.19 μM	–	^236^
			Acetaminophen	1–200 μM	0.21 μM		
Protopasta	–	Electrodeposited Au	Hg^2+^	0–40 μg L^–1^	0.52 μg L^–1^	Bottled water, fish oil	^78^
Black Magic	Chemical (DMF)	Electroplating Cu or Ni	Glucose	1–9 mM	–	–	^237^
Black Magic	Mechanical and Chemical (DMF)	Dropcast GOx	Glucose	0.5–6.3 mM	15 μM	Blood plasma	^77^
			Uric acid	0.5–250 μM	0.02 μM	Saliva, urine	
			Nitrite	0.5–250 μM	0.03 μM	Saliva, urine	
Protopasta	Mechanical and Electrochemical (NaOH)	–	Cu^2+^	10–300 μg L^–1^	0.097 μg L^–1^	Bioethanol	^238^
Black Magic	Mechanical and Chemical (DMF)	Electrodeposition PB	H_2_O_2_	1–700 μM	0.56 μM	Milk	^239^
Black Magic	Chemical and Electrochemical (DMF)	Incubation AuNPs and HRP	H_2_O_2_	0–100 μM	9.1 μM	Human plasma	^240^
				150–600 μM			
Protopasta			Acetaminophen	0–175 mg L^–1^	0.43 mg L^–1^	Urine	^125^
			Caffeine	0–35 mg L^–1^	0.39 mg L^–1^		
Black Magic	–	–	Hg^2+^	0–24 ppb	1.2 ppb	Tap water	^241^
		Bi	Pb^2+^	16–104 ppb	4.1 ppb		
		Bi	Cd^2+^	9–56 ppb	2.2 ppb		
Protopasta	Mechanical and Electrochemical (NaOH)	–	Acetaminophen	0.2–4 μM	0.014 μM	Pharmaceuticals	^242^
Protopasta	Electrochemical	–	Cd^2+^	0.04 – 0.89 μM	9 nM	Wine	^243^
	Fenton		Pb^2+^	0.02–0.48 μM	6 nM		
LATIOHM	Chemical (DMF) Electrochemical (PBS)	–	Dopamine	0–100 μM	1.45 μM	–	^244^
Protopasta	Plasma		Dopamine	M	0.01 μM	Saliva	^80^
			Nitrite	20–300 μM	0.86 μM		
Protopasta	Mechanical and Electrochemical (NaOH)	Antibodies/BSA	Hantavirus	30–240 μg L^–1^	22 μg ^–1^	Human serum	^79^
Black Magic	Mechanical and Electrochemical (NaOH)		Atropine	5–60 μM	1 μM	Beverages	^245^
Black Magic	Mechanical and Electrochemical (PBS)		Cocaine	20–100 μM	6 μM	–	^246^
Protopasta	Mechanical		Nitrite	8–200 μM	2.39 μM	Well water	^247^
						IC validated	
Black Magic	Chemical and Electrochemical (DMF)	GOx	Glucose	50–500 μM	2.97 μM	Apple cider	^248^
Black Magic	Chemical (DMF)		4-nitrophenol	–	–	–	^249^
Black Magic	Chemical (NaOH) Electrochemical (PBS)		L-methionine	5–3000 μM	1.39 μM	Human serum	^250^
Protopasta		Cholesterol or Choline oxidase	Cholesterol	30–240 μM	3.36 μM	Artificial blood	^251^
			Choline	0.5–4 μM	0.08 μM		
Protopasta	Laser		Cd^2+^	25–125 μg L^–1^	1.34 μg L^–1^	–	^252^
			Pb^2+^		1.32 μg L^–1^		
			Cu^2+^		0.31 μg L^–1^		
CB/PLA	Mechanical and Electrochemical (NaOH)		Catechol	0.5–60 μM	0.01 μM	Milk	^253^
			Hydroquinone	1–50 μM	0.22 μM		
Protopasta	Electrochemical Fenton		Epinephrine	5–40 μM	0.61 μM	Artificial urine	^81^
Protopasta	Mechanical and Electrochemical (NaOH)		Naproxen	2–50 μM	0.9 μM	Pharmaceuticals, tap water	^254^
Protopasta	Chemical (DMF) Elecrochemical (NaOH)		β-estradiol	2.5–50 μM	4.18 μM	Synthetic urine	^255^
			Progesterone	12.5–75 μM	17.8 μM		
Protopasta	Plasma Electrochemical (H_2_SO_4_)	GOx	Glucose	1–7 mM	–	–	^256^
Protopasta	–	Dropcast Fe(III)/nafion	Glucose	25–500 μM	4.3 μM	Artificial sweat	^257^
Protopasta	Mechanical w/ alumina and Electrochemical (NaOH)		Dopamine	0.1–2 mM	102 nM	–	^258^
Black Magic	Chemical (DMF) Electrochemical (PBS)		Bisphenol A	0.5–40 μM	0.23 μM	Water, pea water, pea packaging	^98^
Protopasta	Mechanical and Electrochemical (NaOH)	–	Hydroxychloroquine	0.4–7.5 μM	0.04 μM	Pharmaceuticals, water	^259^
Black Magic		AuNP/cDNA	SARS-CoV-2	1–50 μM	0.3 μM	Synthetic saliva, human serum	^260^
Black Magic	Chemical (DMF) Electrochemical (PBS)	Electrodeposited PB	L-cysteine	3–230 μM	0.86 μM	Blood serum	^261^
Protopasta	Electrochemical (NaOH)		Acetaminophen	5–300 μM	0.54 μM	Pharmaceuticals	^262^
Protopasta	Electrochemical (NaOH)		Dopamine	0–10 μM	0.8 μM	–	^145^
PLA/Gt	–	–	Tetracycline	0.5–50 μM	0.19 μM	Tap water, milk	^263^
Protopasta	Electrochemical (NaOH)	–	Creatinine	0.5–35 mM	37.3 μM	Urine	^264^
PLA/Gt	–	–	TNT		0.52 μM	Seawater, river water, tap water	^265^
PLA/Gt	Mechanical and Electrochemical (NaOH)		Ciprofloxacin	2–32 μM	1.79 μM	Pharmaceuticals, milk	^266^
Protopasta	Mechanical		Paraquat	0.5–6 μM	0.19 μM	Tea	^267^
			Pb^2+^	2.5–25 μM	1.1 μM		
			Caffeic acid	3.5–100 μM	2.6 μM		
Black Magic	Chemical (DMF) Electrochemical (PBS)		Dopamine	0.01–20 μM	3 nM	Hydrochloride injection	^268^
Protopasta	Mechanical and Electrochemical (NaOH)		Sulfanilamide	1–10 μM	0.26 μM	Honey	^269^
Protopasta	Mechanical and Electrochemical (NaOH)		Tyrosine	1–250 μM	0.25 μM	Urine	^270^
Protopasta	Electrochemical (NaOH)		Resorcinol	5–400 μM	3.4 μM	River, mine, well and tap water	^271^
PLA/Gt	Mechanical		Paraquat	0.05–1 μM	0.01 μM	Honey, tap and lake water, milk, orange juice	^272^
			Carbendazim	0.05–50 μM	0.03 μM		
Black Magic	Chemical (DMF) Electrochemical (PBS	AuNPs/COF	Acetaminophen	0.1–100 μM	0.076 μM	Pharmaceuticals	^273^
Protopasta		Antibodies/Cd/Se ZnS QDs	Salmonella	10^–3^–10^–5^ cfu mL^–1^	5 cfu mL^–1^	Chicken	^274^
Protopasta	Electrochemical (NaOH)		L-dopa	24–300 nM	–	Human sweat	^275^
Protopasta			Colchicine	0.6–2.2 μM	0.11 μM	Pharmaceuticals	^276^
PLA/Gt	Mechanical and Electrochemical (NaOH)		Atorvastatin	1–200 μM	0.13 μM	Pharmaceuticals	^277^
Protopasta	Electrochemical (NaOH)		Ascorbic acid	0.05–2 mM	13.6 μM	Pharmaceuticals (UV–vis validated)	^64^
			Acetaminophen	5–300 μM	4.5 μM		
rPLA/CB/TTM	Electrochemical (NaOH)		Atropine	5–100 μM	0.51 μM	Gin, whiskey	^278^
rPLA/CB/Gt/CO	Electrochemical (NaOH)		Oxalate	10–500 μM	5.7 μM	Synthetic urine	^161^
Black Magic	Chemical (DMF)		Epinephrine	4–100 μM	0.2 μM	Human serum	^279^
PLA/CB	Mechanical	Antibodies/BSA	SARS-CoV-2	0.01–4.5 nM	2.7 pM	Human serum, synthetic saliva	^280^
rPLA/CB/PES	Electrochemical (NaOH)		Caffeine	1–500 μM	0.23 μM	Tea, coffee	^84^
rPLA/CB	Electrochemical (NaOH)		Acetaminophen	1–300 μM	3.2 μM	–	^83^
rPLA/PES/CB/ MWCNT-COOH	Chemical (NaOH)	Antibodies/BSA	Yellow Fever	0.5–15 μM	0.14 μM	Human serum	^166^
rPLA/CB/CO	Electrochemical (NaOH)		Bisphenol A	0.5–10 μM	0.1 μM	Bottle and tap water	^82^
Protopasta	–	–	Methyl parathion	0.85–19.6 μM	0.2 μM	Honey	^131^
PLA/CB			Carbendazim	0.5–40 μM	0.09 μM	Honey	^281^
Protopasta			Flunitrazepam	2–16 mg L^–1^	0.07 mg L^–1^	Whiskey, vodka, gin, beer	^282^
			Scopolamine	2–26 mg L^–1^	0.56 mg L^–1^		
			Ketamine	0.5–4 mM	0.11 mg L^–1^		
			Acetaminophen	2–16 mg L^–1^	0.51 mg L^–1^		
Protopasta		Electropolymerized Poly(methylene blue)	Uric acid	50–100 μM	9.61 μM	–	^283^
Protopasta	Chemical (NaOH) Electrochemical (BR Buffer)		Phloridzin	5–25 μM	2.2 μM	Juices	^284^
Protopasta	Electrochemical (NaOH)		Serotonin	2–10 μM	0.36 μM	Colonic tissue	^129^
FilaFlex	Mechanical	Dropcast CB	Free chlorine	0.2–20 ppm	0.04 ppm	Tap water	^285^
Protopasta	Chemical (DMF)		Theophylline	5–150 μM	1.2 μM	Artificial urine, pharmaceuticals	^286^
Protopasta	Mechanical and Electrochemical (NaOH)		Flunixin		0.3 μM	Pharmaceuticals	^287^
Protopasta	Electrochemical Fenton and mechanical		Folic acid	10–200 μM	5.1 μM	Juice samples	^288^
Black Magic	Chemical (NaOH) Electrochemical (PBS)	AuNPs/PEDOT:PSS	Chlorogenic acid	10–400 μM	4.13 μM	Coffee powder	^289^
Protopasta/Ag ink	Mechanical and Laser	Dropcast GOElectrodeposited TLGNS	Lamotrigine	0.01–300 μM	0.01 nM	Urine	^290^
Protopasta	Electrochemical(NaOH)		Gallic acid	20–1000 μM	0.17 μM	Wine, tea, water	^291^
PLA/Gt	Mechanical		Amoxicillin	4–12 μM	0.8 μM	Urine, saliva, plasma	^292^
			Acetaminophen	4–12 μM	0.51 μM		
PLA/CB/rGO	Mechanical and Electrochemical (NaOH)		TNT	10–100 μM	0.22 μM	–	^293^
			Cocaine	40–110 μM	2.1 μM		
PLA/CB/PEG/ Bi_2_O_3_ or CuO			Pb^2+^	0–90 μg L^–1^	0.39 μg L^–1^	Artificial blood	^85^
			Cd^2+^	0–90 μg L^–1^	0.68 μg L^–1^		
			Glucose	0.1–1 mM	0.18 mg dL^–1^		
			Uric acid	10–100 μM	1.1 μM		
Protopasta	Blue Laser and Electrochemical (NaOH)		Hydroxychloroquine	0.2–12.3 μM	0.01 μM	Tap water, pharmaceuticals	^117^
			Acetaminophen	1–30 μM	0.2 μM		
PLA/Gt/AgNPs	Mechanical		Pyridoxine	0.1–400 μM	0.03 μM	Pharmaceuticals	^294^
Protopasta	Laser	Au	Glucose	0.039–15.2 mM	10.6 μM	Rehydration drinks	^118^
Protopasta	Chemical (DMF)Electrochemical (PBS)	AuNPs	Dopamine	0.01–20 μM	0.084 μM	Pharmaceuticals	^295^
PLA/Gt	Mechanical		25E-NBOH	0.85–5.1 μM	0.2 μM	Blotting Paper	^296^
PLA/Gt/C_3_N_4_	Mechanical		Amaranth dye	0.2–4.2 μM	0.06 μM	Food and drink	^297^
Protopasta	Mechanical		Sulfanilamide	1.99–10.78 μM	0.2 μM	Tap water, wastewater	^298^
			Ciprofloxacin	4.96–40.61 μM	0.6 μM		
Black Magic	Chemical (NaOH)	AuNPs/COF	Methotrexate	0.5–100 μM	0.07 μM	Pharmaceuticals	^299^
Protopasta	Electrochemical (NaOH)	PB	H_2_O_2_	2–1000 μM	0.4 μM	Cell cultures	^300^
Protopasta	Plasma pen		Capsaicin	0.01–1 μM	0.003 μM	Sauces	^113^
				2–6 μM			
rPETg/CB/Gn/MWCNT	Electrochemical (NaOH)		Uric acid	30–500 μM	0.27 μM	Synthetic urine	^86^
			Nitrite	0.1–5 mM	2.69 μM		
PP/CB	Electrochemical (NaOH)		Colchicine	2–50 μM	10 nM	Tap, bottle and river water	^27^
rPLA/CO/CB/MWCNT	Electrochemical (NaOH)		Acetaminophen	5–60 μM	0.04 μM	Pharmaceuticals	^165^
			Phenylephrine	5–200 μM	0.38 μM		
PLA/Gt/Al_2_O_3_	Mechanical		Sulfamethoxazole	2–40 μM	0.4 μM	Honey	^301^
rPLA/CO/CB/Gt	Electrochemical (NaOH)		β-estradiol	0.04–1.5 μM	21 nM	Tap, bottle, river, lake water	^162^
rPLA/CO/CB	Mechanical	Antibodies/BSA	Human Monkeypox	0.01–1 μM	2.7 nM	Human serum	^302^
rPLA/CO/CB/Gt	Mechanical		TNT	5–20 μM	0.88 μM	Surfaces	^114^
Protopasta	Chemical (NaOH)Electrochemical (NaOH)		Fentanyl	0.1–9.1 μM	0.017 μM	Synthetic urine	^303^
PLA/rGO/CB/PB-Nb_2_O_5_	Mechanical		Nitrite	5–500 μM	0.92 μM	River water, lake water	^304^
Electrifi	Laser	–	H_2_O_2_	0–300 μM	35.22 μM	–	^305^
Protopasta	Electrochemical (NaOH)	–	Ascorbic acid	0–5 mM	0.13 mM	Fruit	^306^
PLA/PP/CB	MechanicalElectrochemical (NaOH)		Quercetin	2–80 μM	0.56 μM	Honey	^307^
PLA/CB	Mechanical Electrochemical (NaOH)		Ciprofloxacin	1–12.5 μM	0.3 μM	Tap water, synthetic urine	^308^
Protopasta	Mechanical Electrochemical (NaOH)		Acetaminophen	0.41–48.61 μM	0.44 μM	Pharmaceuticals	^309^
			Caffeine	0.87–102.93 μM	0.58 μM		
Black Magic	Blue laser		Acetaminophen	5–50 μM	0.5 μM	–	^119^
PLA/Gt/Ni	MechanicalElectrochemical (NaOH)		Glucose	0.05–1.5 mM	2.6 μM	Synthetic saliva, blood plasma	^310^
Protopasta	Chemical (DMF) Electrochemical (NaOH)	Au/NGQDs	Dopamine	0.01–30 nM	9.4 pM	Pharmaceuticals	^311^
Protopasta	Electrochemical (NaOH)		Pyrogallol	1–150 μM	0.4 μM	Mine, river, tap water	^312^
Protopasta	Mechanical Electrochemical (NaOH)		Acetaminophen	0.5–23 μM	0.21 μM	River, groundwater, lake, tap water	^313^
			Diclofenac	0.2–1.2 μM	0.051 μM		
Anycubic PLA	Chemical (acetone)		L-tryptophan	15–70 μM	5 μM	Food additives, HPLC verified	^314^
PLA/CB/CO/Biochar	Electrochemical (NaOH)		Carbendazim	0.1–5 μM	0.01 μM	Lake and tap water, lemon juice	^185^
PLA/CB/Babassu Oil			Cocaine	5–200 μM	1.2 μM	Seized Samples	^174^
PLA/CB/Soy Bean Oil	Mechanical Electrochemical (NaOH)		Catechol	3–40 μM	0.7 μM	River and tap water	^175^
PLA/Gt/Al_2_O_3_	Mechanical		TNT	0.5–6 μM	0.071 μM	Surfaces, Tap, lagoon, seawater	^315^
Protopasta	Plasma		Methyldopa	0.01–100 μM	7 nM	PharmaceuticalsRiver water	^316^
Protopasta	Mechanical Electrochemical (NaOH)		Bithionol	0.03–5.7 μM	11.54 nM	River water, synthetic urine	^317^
Protopasta	Chemical (NaOH)		Nimesulide	1–75 μM	0.19 μM	Sewage samples	^318^
Protopasta	Mechanical Electrochemical (NaOH)	Ag Particles	Nitrate	5–80 mg L^–1^	2.7 mg L^–1^	Tap, sea, aquarium water	^319^
Protopasta		PB + AOx	Ethanol	0–100 mM	0.4 mM	Wine	^127^
		PB + GOx	Glucose	0–2 mM	9.5 μM		
Protopasta	Mechanical		Nicotine	4.9–162.2 mg L^–1^	2.7 mg L^–1^	e-cigarettes	^320^
PLA/PEG/CB CuO			Glucose	0–1 mM	5.1 μM	–	^169^
			Lactic acid	0–13.75 mM	0.12 μM		
			Creatinine	0–40 μM	1.5 μM		
Protopasta	Blue laser	Co^2+^	Glucose	50–400 μM	6.3 μM	Synthetic urine	^321^
Protopasta	Electrochemical (NaOH)		Secnidazole	2.5–250 μM	0.2 μM	Pharmaceuticals	^322^
TPU/CB	Electrochemical (NaOH)		Dopamine	0.1–0.6 μM	11 nM	Synthetic urine	^28^
			Uric acid	10–60 μM	0.53 μM		
			Nitrite	10–60 μM	8.4 μM		
PLA/CO/CB/Bi_2_O_3_	Electrochemical (NaOH)		Pb^2+^	0–300 μg L^–1^	0.79 μg L^–1^	Atmospheric water	^168^
TPU/CB/Gt	Mechanical Electrochemical (NaOH)		Uric acid	2.5–100 μM	1.3 μM	Synthetic sweat	^164^
rPP/CB	Electrochemical (NaOH)		Acetaminophen	5–40 μM	0.14 μM	Synthetic urine	^90^
			Phenylephrine	5–40 μM	0.04 μM		
			Uric acid	5–60 μM	0.03 μM		
rPLA/CO/CB/microcellulose	Electrochemical (NaOH)		Dopamine	0.01–1 μM	^3 nM^	Human serum	^323^
Protopasta	Electrochemical (NaOH)		Nitrite	5–500 μM	1.8 μM	Synthetic saliva, river, mine, tap, well water	^324^
Protopasta	Electrochemical (NaOH)		Amoxicillin	0.1–5 μM	10 nM	Tap, drinking, river, lake water	^325^
rPLA/CO/CB/Gt-AgNPs	–	–	Cd^2+^	1.5–100 μg L^–1^	0.43 μg L^–1^	River water	^87^
rPS/Gt	Mechanical		Sulfanilamide	1–10 μM	0.3 μM	Water, milk, otologic solution	^326^
				10–50 μM			
rPLA/CO/CB/MWCNT	Electrochemical (NaOH)		Carbendazim	5–40 μM	0.26 μM	River water	^148^
rPLA/CO/CB/Gt-AuNPs	Electrochemical (NaOH)		Pb^2+^	1–75 μg L^–1^	0.89 μg L^–1^	River water	^88^
rPLA/CO/CB/Biochar	Electrochemical (NaOH)		Carbendazim	0.2–40 μM	57 nM	River, lake water	^91^
PP/CB/Gt	Electrochemical (NaOH)		Chlorpromazine	0.1–1.2 mM	80 μM	–	^327^
rPLA/CO/CB/Gt	Electrochemical (NaOH)		Morphine	0.5–10 μM	0.05 μM	Pharmaceuticals	^328^
			Codeine	1–10 μM	0.07 μM	Synthetic salvia	
PP/CB	Electrochemical (NaOH)		Atropine	0.65–20.83 mg mL^–1^	0.15 mg mL^–1^	Saliva, urine, vitreous humor, beverages	^329^
rPLA/CO/CB/Gt	–	–	Ketamine	10–250 μM	0.7 μM	Wine, beer, water, vodka	^330^
rPLA/CO/CB/Gt	Mechanical		Capsaicin	5–20 μM	1.21 μM	Sauces	^331^
Protopasta	Electrochemical (Acetate)	PB or AOx or Lox	Glucose	0–200 μM	4.3 μM	Sweat	^126^
			Alcohol	0–36 mM	0.8 mM		
			Lactic acid	0–40 mM	0.6 mM		
			Uric acid	0–50 μM	1.5 μM		
			Caffeine	0–50 μM	1.2 μM		

a **Key**: PBS – phosphate buffer solution; TNT – 2,4,6-trinitrotoluene; NADH – nicotinamide adenine dinucleotide; DMF – dimethyl formamide; Gox – glucose oxidase; PB – prussian blue; AuNPs – gold nanoparticles; HRP – horseradish peroxidase; BSA – bovine serum albumin; IC – ion chromatography; COF – covalent organic framework; TTM – tris­(2-ethylhexyl) trimellitate; CO – castor oil; PES – poly­(ethylene succinate); rPLA – recycled PLA; PEDOT:PSS - poly­(3,4-ethylenedioxythiophene):poly­(styrene sulfonate); TLGNS - horn-like gold nanostructure; AgNPs – silver nanoparticles; COF – covalent organic framework; rPETg – recycled poly­(ethylene terephthalate glycol); Gn – graphene; PP – poly­(propylene); NGQDs – N-doped graphene quantum dots; AOx – alcohol oxidase; rPS – recycled poly­(styrene); Lox – lactate oxidase;

**Figure 14:**
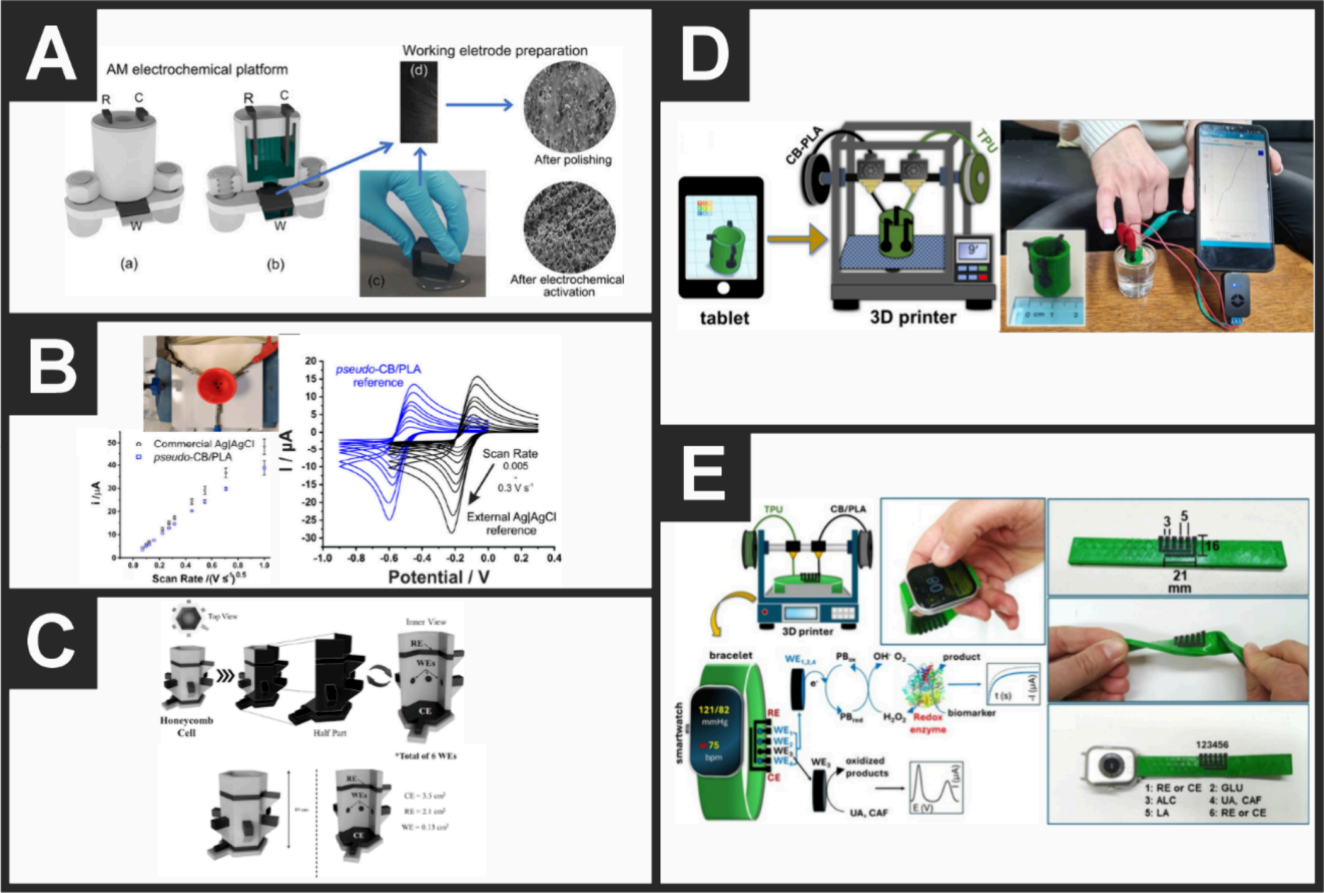
(A) Image of one of the first reported fully additively manufactured electrochemical cells, which developed the most popular activation method to date. Reproduced with permission from ref 74. Copyright American Chemical Society 2019. (B) Photograph and electrochemical performance of an all-in-one printed electroanalytical cell using a dual-extruder printer. Reproduced with permission from ref 64. Copyright American Chemical Society 2021, license CC-BY 4.0. (C) Images of a honeycomb designed cell, with 6 working electrodes and shared counter and reference electrodes, produced in a single print. Reproduced with permission from ref 131. Copyright American Chemical Society 2023, license CC-BY 4.0. (D) Schematic illustration of the production steps of the 3D printed wearable e-finger and photograph of its use. Reproduced with permission from ref 282. Copyright American Chemical Society 2022. (E) Schematics for the production and sensing mechanisms, as well as photographs of printed sensors for a 3D printed biobracelet. Reproduced from ref 126. Copyright American Chemical Society 2025, license CC-BY 4.0.

In this work, the authors print the cell components from a commercial nonconductive ABS filament as this polymer offers better chemical stability compared to PLA. The electrodes were then printed with Protopasta conductive PLA, with the reference painted with a silver ink to create a *pseudo* reference electrode. After printing, the working electrode was treated mechanically and electrochemically, first reported the most commonly used activation procedure still used today, where the electrode is placed within NaOH (0.5 M) and subjected to + 1.4 V for 200 s, followed by −1.0 V for 200 s.^74^ After this, the cell was assembled, using a rubber O-ring to ensure the electrochemical cell was water tight. This cell was used for the detection of dopamine, but the primary novelty lies in the activation procedures and proof of full cell printing.

To remove the necessity for postprint assembly of cells, researchers moved to the use of multiextrusion printer systems. Figure 14B shows one of the first reports of this, using Protopasta to print identical electrodes within a nonconductive PLA cup.^64^ The electrodes were connected to stems on the outside of the cup for ease of connection, and the electrodes were kept at the same size, removing any confusion in attachment, however this is not the most optimal set up for electrochemical performance. To push this concept further, Figure 14C shows a cell printed all in one, again with Protopasta as the electrodes and nonconductive PLA for the cell, but with 6 separate working electrodes.^131^ The honeycomb design allowed for the printing of a working electrode on each wall, with the base of the cell acting as the counter electrode as it has a large surface area, and a ring printed for the reference electrode. In this way, a measurement for a single analyte can be performed 6 times, maximizing reliability, or 6 different analytes could be observed using different methods for each electrode, or any combination in between. These novel designs show what can be produced in-house due to the rapid prototyping and low-cost nature of additive manufacturing.

Other novel device designs can be seen in Figure 14 D and E, where a ring system for the detection of analytes in spiked drink samples,^282^ and a wearable bracelet for biomonitoring have been developed.^126^ In both cases, the wearable parts were produced from nonconductive TPU to offer excellent flexibility, and the electrodes printed from Protopasta. This combination is viable and works well due to the good synergy between TPU and PLA, offering good adhesion between the materials. This is not the case with all materials and careful consideration to this should be made in the design phase of new devices. It is works such as these that push the boundaries of cell designs and applications, where the use of commercial conductive filament is still beneficial. In these works, the relatively poor performance of the conductive filament does not limit the systems, but also help the generate new ideas within the field for future works when filament performance is improved.

The development of novel cells and designs has also been seen in work where bespoke filaments are produced to improve the performance in conjunction with the designs. This can be seen in work from Kalinke et al.^166^ where a novel full electroanalytical biosensing cell was produced for the detection of Yellow Fever Virus cDNA, Figure 15A. The authors created a bespoke filament from rPLA (65 wt%), with PES (10 wt%), CB (15 wt%) and carboxylated MWCNT (10 wt%). The electrodes showed significant electrochemical improvements compared to electrodes printed from commercial filament, with an approximately 3 times larger heterogeneous electron charge transfer rate constant (*k* ^*0*^) and a 3.5 times larger electrochemically active surface area. Additional benefits for the creation of a biosensor were seen through the use of carboxylated MWCNTs, which readily offered increased amounts of immobilization sites for the capture DNA. Through the unique benefits of additive manufacturing the authors developed a cell, where the additive manufactured reference and counter electrodes were embedded within the lid of the cell, which could be reused. The working electrode could be removed and replaced for each sample, ensuring no cross-contamination. An additional lip was included in the design, as seen in Figure 15A, to allow for reproducible construction of the biosensing platform through the addition of stable droplets, this ensured that the wider cell was not contaminated, or reagents wasted. This work shows a high-end application for additive manufacturing electrochemistry, but due to the low-cost nature of FFF and electrochemical technology, home applications are a distinct possibility in the future. To this end a similar bespoke filament with rPLA (65 wt%), castor oil (10 wt%), CB (15 wt%) and MWCNT (10 wt%) was reported for the simultaneous detection of acetaminophen and phenylephrine within home cold and flu pharmaceuticals.^165^ In this work the electrodes and holder were designed to go over a mug, Figure 15B, allowing for reproducible home sensing in drinks. Impressively, this filament showed an almost 6 times improvement in *k* ^*0*^ value compared to commercial filament, showing both the benefits of bespoke filament and cell design.

**Figure 15:**
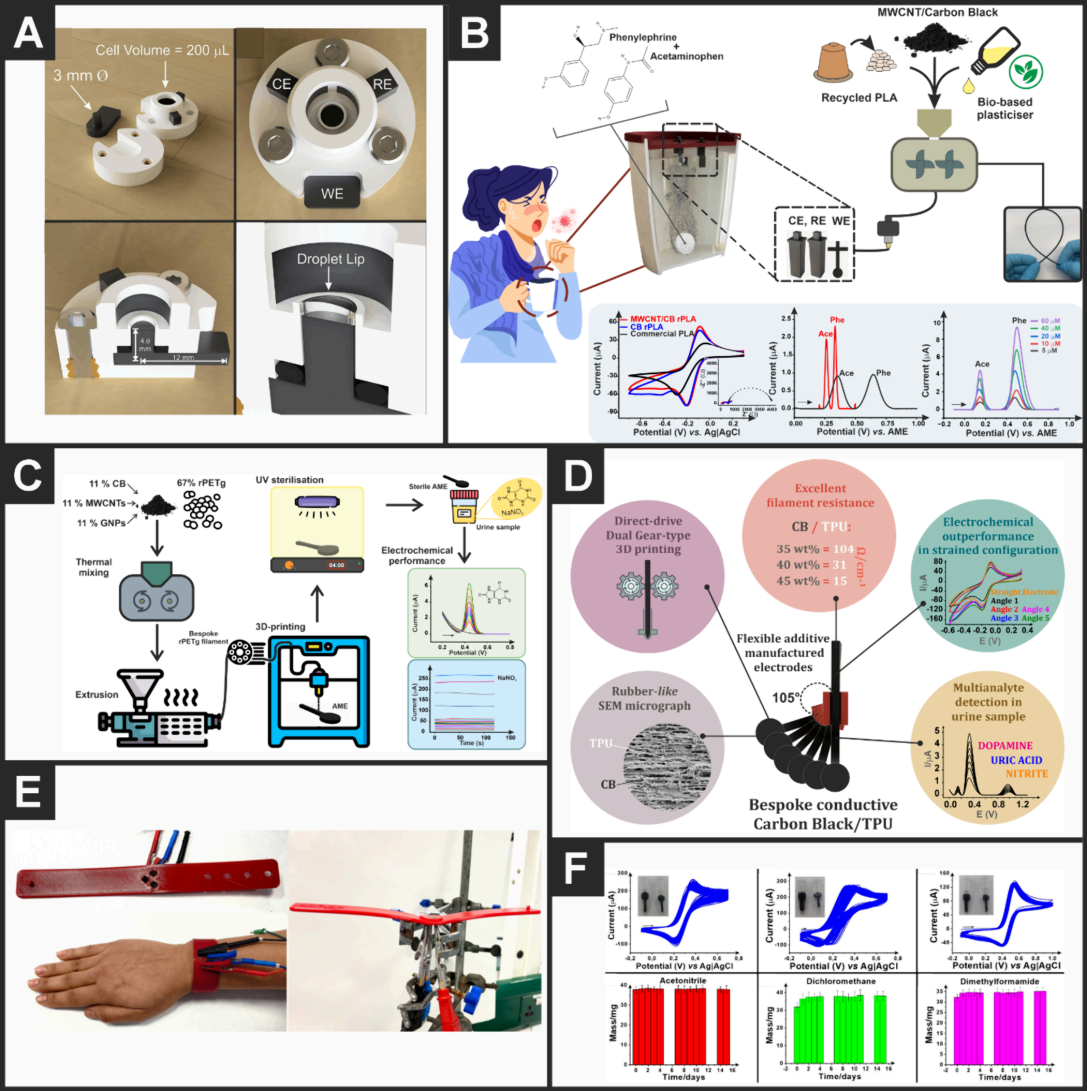
(A) Render images of the additive manufactured cell for the detection of Yellow Fever Virus cDNA. Reproduced with permission from ref 166. Copyright Elsevier 2023, license CC-BY 4.0. (B) Schematic showing the filament production, print at home device, and electrochemical response of the rPLA/CO/CB/MWCNT filament for acetaminophen and phenylephrine detection. Reproduced from ref 165. Copyright Springer Nature 2024, license CC-BY 4.0. (C) Schematic of the filament production, printing, sterilization, and performance of a conductive rPETg filament. Reproduced with permission from ref 86. Copyright Elsevier 2024, license CC-BY 4.0. (D) Summary of the first high loading conductive TPU for sensors, showing flexibility and performance. Reproduced with permission from ref 28. Copyright Elsevier 2025, license CC-BY 4.0. (E) Photograph of the additive manufactured flexible bracelet sensor with bespoke conductive TPU electrodes. Reproduced with permission from ref 164. Copyright American Chemical Society 2025, license CC-BY 4.0. (F) Photographs, 100 cycle CVs and the change in weight over 15 days for PP printed electrodes in acetonitrile, dichloromethane, and dimethylformamide. Reproduced with permission from ref 27. Copyright American Chemical Society 2024, license CC-BY 4.0.

Crucially for sensing applications, due to the ingress of solutions in the polymer matrix,^332^ the electrodes printed from PLA-based filament remain single-use items as to avoid cross-contamination between samples. To counter issues such as this and produce electrodes with enhanced chemical stability and reuse potential, researchers have recently reported a range of new conductive filaments with different base-polymers. The first report was the production of conductive PETg,^167^ a common material already used within FFF printing, where researchers used a combination of CB, Gn and MWCNT to create an optimal conductive network through the insulating polymer matrix. Figure 15C shows an example of the workflow from a work producing this filament with the aim of detecting uric acid and nitrite within healthcare settings.^86^ This filament was created from recycling waste PETg filament/prints as the base polymer (67 wt%), with equal amounts of CB (11 wt%), Gn (11 wt%), and MWCNT (11 wt%), through a thermal method. Importantly, unlike commercial PLA, electrodes printed from rPETg filaments have been shown to be sterilizable, both through alcoholic and UV methods, without this affecting their electrochemical performance.^86^ In addition to this, the electrode was shown to be reuseable up to 10 times before a reduction in performance, giving an indication this material could lead to significantly less waste. Although a promising step forward, this material did not have the same flexibility or conductivity of many of the bespoke PLA filaments that have been reported. More work needs to be done to bring this conductive material in line.

One bespoke material reported recently that has significant flexibility is conductive TPU.^28,164,333^ Although commercial conductive TPU is available, for example NinjaTek Eel^TM^, the conductivity for this filament is too low for electrochemical applications and is marketed as a static dissipative filament. There was initial work that looked to modify the surface of TPU printed parts through dropcasting CB on the surface,^285^ but a truly conductive TPU filament for electroanalytical applications was first reported with a composition of TPU (60 wt%) and CB (40 wt%).^28^ This work highlighted important insights into 3D printer technology that is required when developing highly conductive bespoke material from new base polymers, and in-particular highly flexible ones such as TPU and TPEs, Figure 15D. In particular, Dual gear direct-drive extruders are recommended for flexible materials due to the shorter filament path between extruder gears and the hot-end, meaning there is a lower possibility of material bunching up on itself and jamming the system. In this work, the authors tested the electrochemical performance of the electrodes under different angular strains ranging from 165 to 105°, showing no deterioration in performance. This indicated strong potential for use within flexible and wearable sensors, which was later shown for a bespoke filament produced from TPU (60 wt%), CB (20 wt%), and Gt (20 wt%).^164^ In this work, the authors report the use a 1:1 ratio of CB and Gt as beneficial to the performance of the material in multiple ways. First, as seen in earlier work with PLA, the inclusion of Gt reduces the material cost of the filament significantly, with the authors stating their Gt costing ∼ 4 times less than CB. Second, due to exploring different postprocessing routines they were able to significantly improve the electrochemical performance. Through mechanical polishing before electrochemically activating the electrodes, they found that instances with Gt significantly improved their performance due to exfoliation of the Gt. This improvement worked up to a 1:1 ratio, whereby above this ratio, the reduction in quality of the conductive pathways through the insulator became the overriding issue. Figure 15E shows an example of a printed flexible bracelet/wristband sensor, where the band is printed from a nonconductive TPU and the electrodes printed from the bespoke conductive TPU. This device was successfully applied to the detection of uric acid in artificial sweat and shows a real example of the potential future for these materials. If we consider other technologies, such as 3D scanning, which synergize well with additive manufacturing, one can imagine the creation of bespoke wearable devices that maximize both data quality and user comfort.

One other base polymer reported for electroanalytical applications is PP. This has been targeted in particular due to its remarkable chemical stability compared to the other materials reported, opening up significant avenues to additive manufacturing electrochemistry. The first conductive PP was reported using a thermal mixing method of PP (60 wt%) and CB (40 wt%), showing a remarkable conductivity and electrochemical performance, with a *k* ^*0*^ of 2.0 × 10^–3^ cm s^–1^ being comparable to the best reports of bespoke conductive PLA.^27^ This work was applied to the detection of colchicine within environmental waters giving excellent performance in aqueous environments, but crucially this material has opened up work within nonaqueous environments for additive manufacturing electrochemistry. In this report, the electrodes were used for electrochemistry within dichloromethane, acetonitrile, and dimethylformamide, Figure 15F, showing excellent chemical stability over the course of 100 scans and immersion over 15 days. To further prove the stability, the electrodes were applied toward electrosynthesis in acetonitrile. This has opened the field of electrosynthetic applications for the field of additive manufacturing, previously incompatible due to the poor stability of base polymers; we expect significant advances in this field over the coming years. Taking conductive PP further, a CB and Gt filament in a 1:1 ratio has been reported as a electroanalytical sensor in acetonitrile for chlorpromazine,^163^ and more recently CB based filaments have been produced for a micro well plate for the detection of atropine in spiked drinks using multiextrusion printing.^329^ In addition to this, recent work has showed the formation of conductive filament from hospital lab waste PP, which again showed excellent stability, being able to be reused up to 50 times without losing performance and also being sterilizable without it affecting the electrochemical performance.^90^ All these works show the promise of conductive PP as a material for use in additive manufacturing electrochemistry as a whole and we foresee this material finding wider use throughout the area in the coming years.

## 6 Challenges and Limitations

Despite its rapidly expanding adoption, additive manufacturing, particularly FFF, still faces several limitations that restrict its effectiveness in electrochemical device fabrication. Foremost among these are the constraints imposed by base polymer selection. Polymers such as PLA, ABS, PETg, PP, and TPU dominate the literature due to their accessibility and processability, yet each presents inherent trade-offs that affect chemical compatibility, structural integrity, and long-term device performance. PLA, while easy to print with excellent dimensional stability, exhibits poor chemical resistance and undergoes hydrolytic degradation, limiting its suitability for aggressive electrolytes or prolonged electrochemical cycling. ABS provides improved toughness but has limited stability in oxidizing or acidic environments and generates hazardous fumes during printing, reducing its practical utility in laboratory environments. PETg offers favorable toughness and ease of printing, but its tendency for moisture uptake and hydrolytic instability can affect both mechanical strength and electrochemical behavior. PP provides excellent chemical resistance, but its high shrinkage, warping behavior, and poor interlayer adhesion complicate reliable fabrication and challenge postprinting functionalization strategies. Flexible materials such as TPU enable stretchable or wearable electrochemical devices but bring significant processing challenges, including moisture sensitivity and extrusion instability, often sacrificing accuracy and reproducibility.

These material-specific limitations are compounded by fundamental constraints of the FFF process itself. The layer-by-layer deposition approach inherently produces feature sizes on the order of hundreds of micrometres, preventing the fabrication of microstructured electrode geometries or fine fluidic networks. Surface roughness, stair-stepping artifacts, and incomplete filament fusion introduce variability in electroactive surface area and consequently in device-to-device electrochemical response. Moreover, reproducibility remains a persistent challenge, influenced by filament diameter fluctuations, nozzle wear, printer calibration, and environmental factors such as humidity, all of which can alter extrusion flow, interlayer bonding, and overall dimensional accuracy. Thermal gradients within the print environment can lead to warping, shrinkage, or delamination, further reducing print fidelity and long-term structural stability. These limitations often necessitate postprocessing interventions to stabilize performance across batches.

Even when moving toward bespoke conductive filaments, critical challenges persist. Achieving consistent dispersion of carbonaceous fillers remains a major bottleneck; localized agglomeration, void formation, and polymer–filler incompatibilities introduce heterogeneity along the filament length and across printed structures. Balancing filler loading with melt rheology is equally problematic: increasing the active material content enhances conductivity but can severely impair filament flexibility, reduce melt flow, and compromise layer adhesion. Furthermore, the field lacks standardized protocols for filament production, postprinting activation, and electrochemical characterization. Divergent activation methods, inconsistent reporting of conductivity, and varied benchmarking practices hinder meaningful comparison across studies and impede the establishment of best-practice guidelines. Long-term stability and environmental durability, especially under repeated cycling, mechanical loading, or exposure to humidity and solvents, remain insufficiently studied, representing a significant barrier to real-world deployment.

Finally, although high-performance polymers such as PEEK, Nylon, polycarbonate, and HDPE offer promising combinations of chemical resilience, mechanical strength, and thermal stability, they remain almost entirely unexplored within additive manufacturing electrochemistry. Their adoption is hindered by limited availability of conductive grades, challenging processing requirements, and incomplete understanding of how their intrinsic properties interact with electrochemical environments. Nonetheless, these engineering polymers represent critical opportunities for expanding additive manufactured electrochemistry into harsh-environment sensing, high-temperature operation, and long-lifetime device platforms.

Overall, the limitations across materials, printing processes, and bespoke filament development reveal that additive manufacturing for electrochemistry is governed by inherent trade-offs. Advancing the field will require coordinated innovation in polymer engineering, dispersion science, process control, and standardized characterization.

## 7 Future Perspectives

The field of additive manufacturing electrochemistry is still in its infancy, and we foresee several key areas of growth in the future of the field, which we outline below:

1 **Further expansion of the conductive material pool** - Future research should continue focus on engineering filaments with intrinsic conductivity and chemical stability, incorporating advanced fillers while maintaining printability.
2 **Expansion into new additive manufacturing techniques** – Material extrusion techniques have dominated the field of additive manufacturing electrochemistry, but the expansion into other areas such as SLS is seen as promising.
3 **Hybrid additive manufacturing** - Combining FFF with high-resolution techniques such as stereolithography (SLA) or inkjet printing could overcome resolution limitations and enable multimaterial architectures. Hybrid systems could allow precise deposition of conductive inks onto 3D-printed substrates, improving reproducibility and device miniaturization.
4 **Expansion into hybrid analytical techniques –** The combination of these systems with other techniques such as fluorescence and luminescence offer the chance to create unique and powerful new tools, in particular for diagnostics and environmental monitoring.
5 **Material Formulation for Harsh Electrochemical Environments** - Research should prioritize polymers and composites that resist aggressive electrolytes while maintaining mechanical robustness. This includes exploring chemically cross-linked systems, and nanocomposite blends tailored for electrochemical stability.
6 **Process Monitoring and Closed-Loop Control** - To address reproducibility challenges, real-time monitoring of extrusion parameters, temperature profiles, and layer adhesion should be implemented. Machine learning-driven feedback systems could optimize print conditions dynamically, reducing variability across batches.
7 **Miniaturization and High-Resolution Printing** - Moving beyond conventional FFF, the adoption of microextrusion or multiaxis printing could enable finer feature sizes suitable for microelectrodes and integrated sensing platforms.
8 **Sustainable and Circular Material Strategies** - With growing emphasis on sustainability, developing more recyclable conductive filaments and low-energy postprocessing methods will be critical for scaling additive manufactured in electrochemical applications. The implementation of true circular economy systems should be sought to reduce the impact of the waste plastic material.

## 8 Conclusions

Additive manufacturing, particularly FFF, has rapidly evolved from a prototyping aid into a transformative technology for electrochemical device fabrication. Its capacity to produce complex architectures, customizable geometries, and rapidly iterated designs has unlocked new possibilities for sensing platforms, energy devices, and wearable systems. This review consolidates and critically evaluates the full breadth of these advances to clarify the current landscape and objectively assess how the field is progressing.

Recent developments in conductive polymer composites, postprinting surface functionalization, and multimaterial printing illustrate that many of the early barriers, including limited conductivity and restricted material availability, are now being systematically addressed. However, fundamental challenges persist. Achieving microscale feature resolution, ensuring batch-to-batch reproducibility, and optimizing the tradeoff between printability and electrochemical performance remain priority areas that require coordinated innovation.

The work presented in this review highlights both the momentum of the field and the critical gaps that must be overcome. Continued progress in electrochemically active filaments, hybrid manufacturing workflows, in situ characterization, and intelligent process control will be essential to establish additive manufacturing as a reliable, scalable, and high-definition fabrication route. With sustained effort in these directions, additive manufacturing electrochemistry is well positioned to deliver chemically robust, high-resolution, and application-tailored solutions that will redefine practical capabilities across electroanalytical science, energy storage, and beyond.
